# Supplementary figures and images for: Gellan gum-based granular gels as suspension media for biofabrication
Source: PLoS One. 2024 Nov 27;19(11):e0312726. doi: 10.1371/journal.pone.0312726 (PMC11602023; doi:10.1371/journal.pone.0312726)

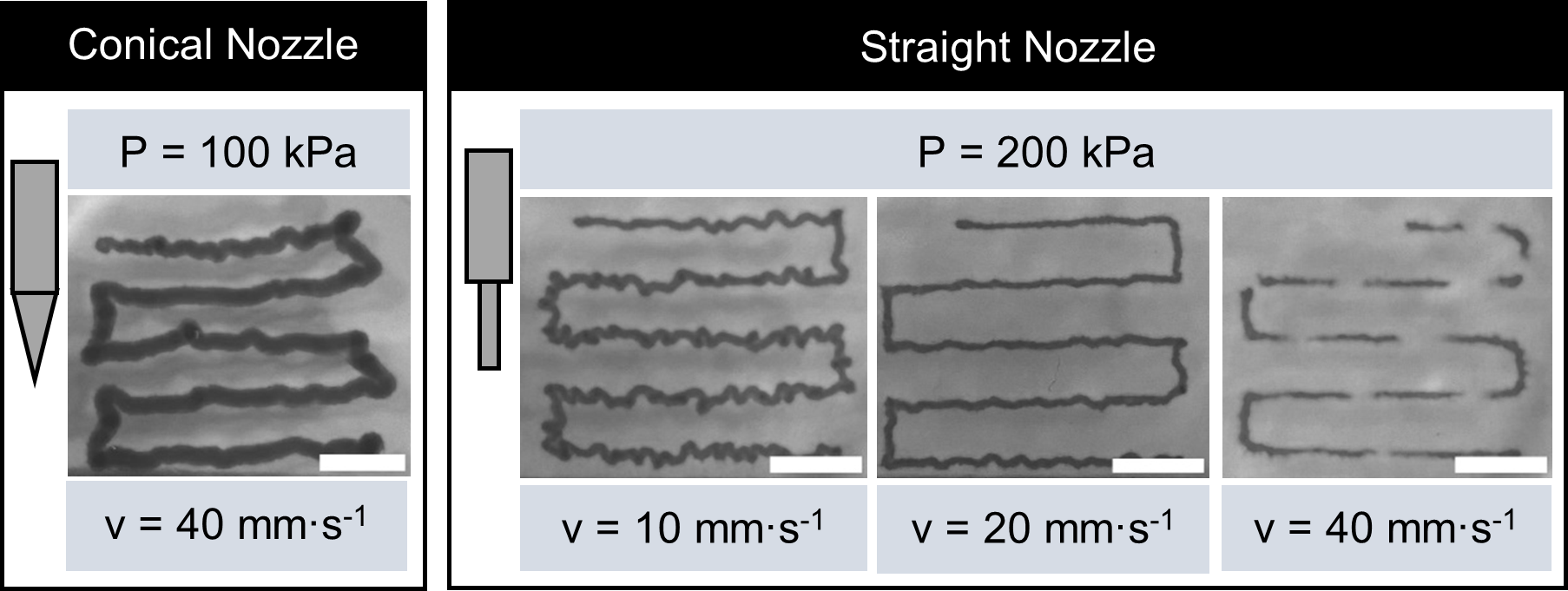

Supplement: S1 Fig — Both the conical and straight nozzles have an inner diameter of 25G. (scale bars: 5 mm). The straight nozzle permitted printing at a high pressure compared to the conical nozzle, negating the over-deposition of ink seen when printing with this nozzle. Printing parameters: ink formulation = 30% w/v poloxamer, print temperature = 30°C. The distance between the nozzle’s tip and the substrate was calibrated to be the thickness of a piece of standard 80 gsm paper. (TIF) [file pone.0312726.s001.tif]

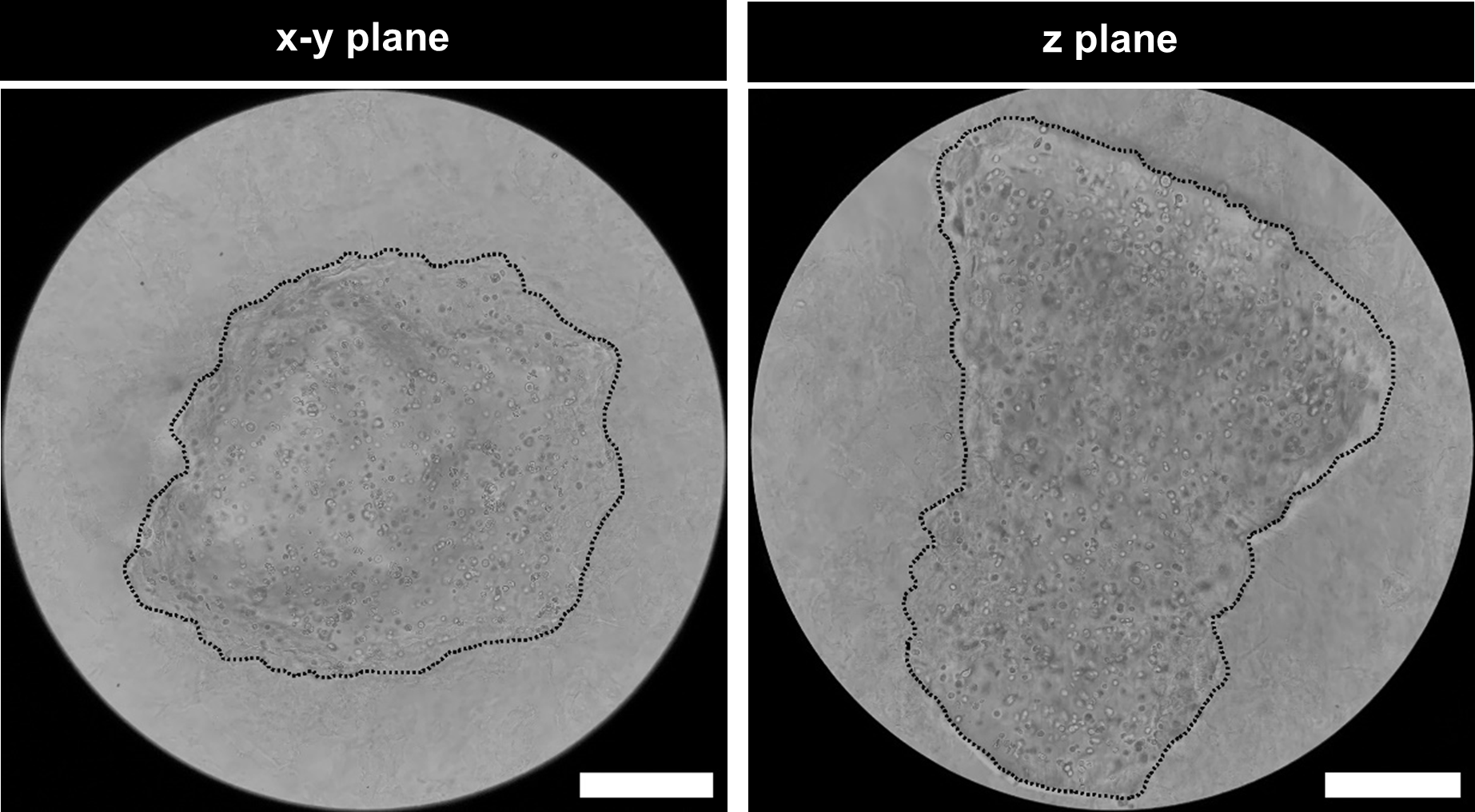

Supplement: S2 Fig — Droplet printed with a gelMA-based bioink. The droplet was photocured in the granular gel, prior to unjamming of granular gel with culture media to permit rotation of the droplet and imaging in the z-plane. (scale bars: 500 μm). (TIF) [file pone.0312726.s002.tif]

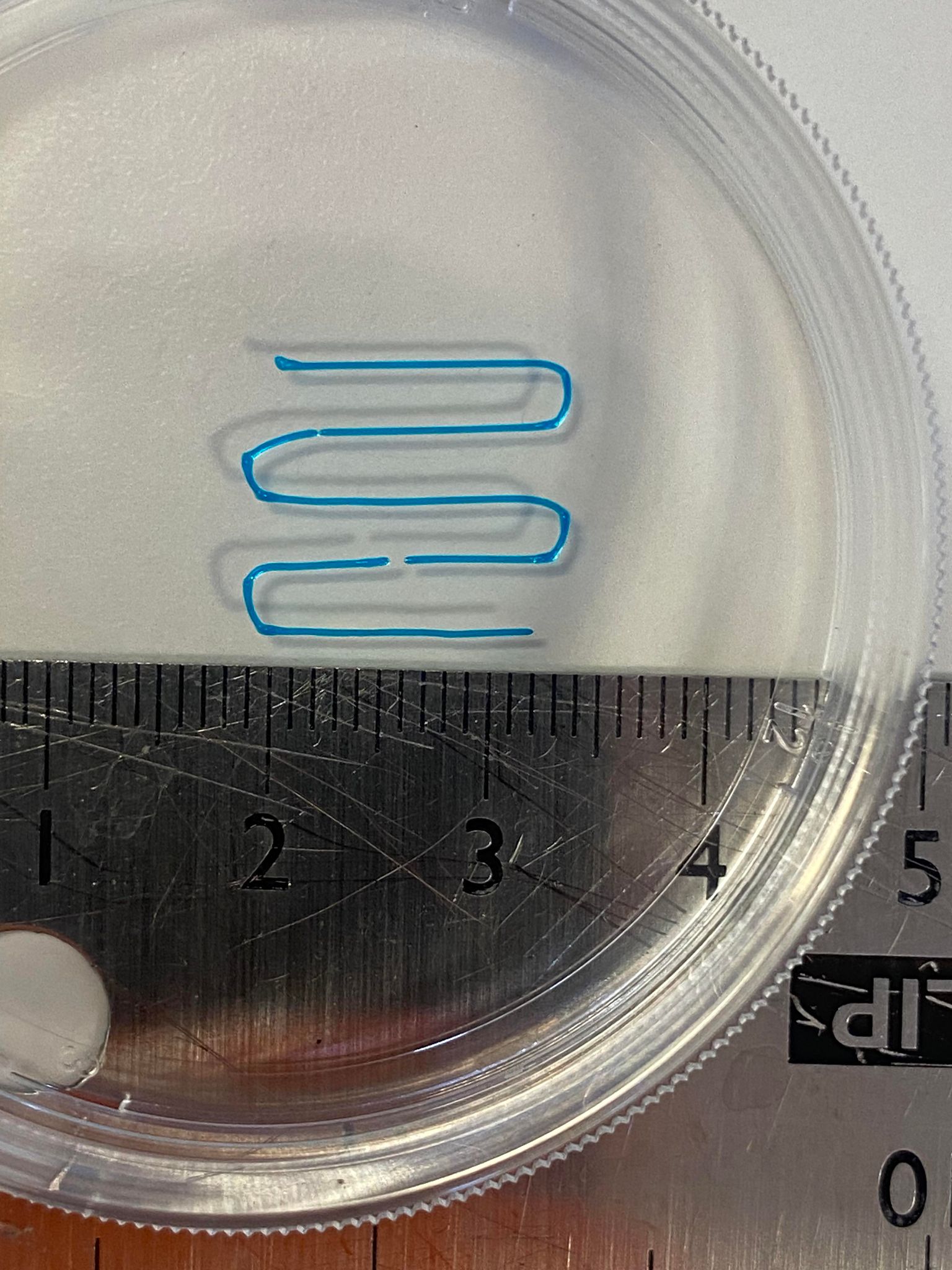

Supplement: S3 Data — (ZIP) [file pone.0312726.s005.zip › Figure 3 data/Filament printing/1.jpg]

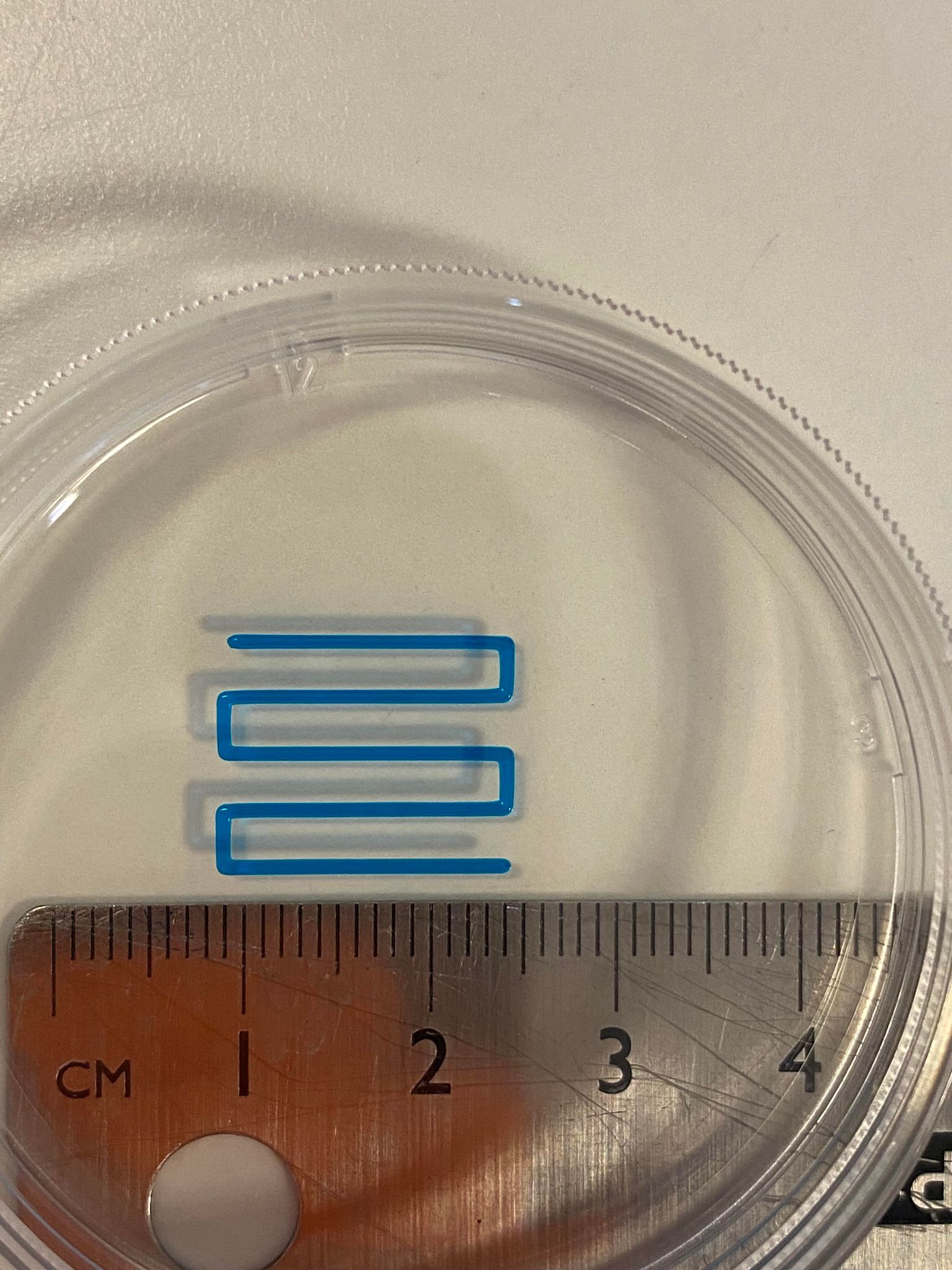

Supplement: S3 Data — (ZIP) [file pone.0312726.s005.zip › Figure 3 data/Filament printing/10.jpg]

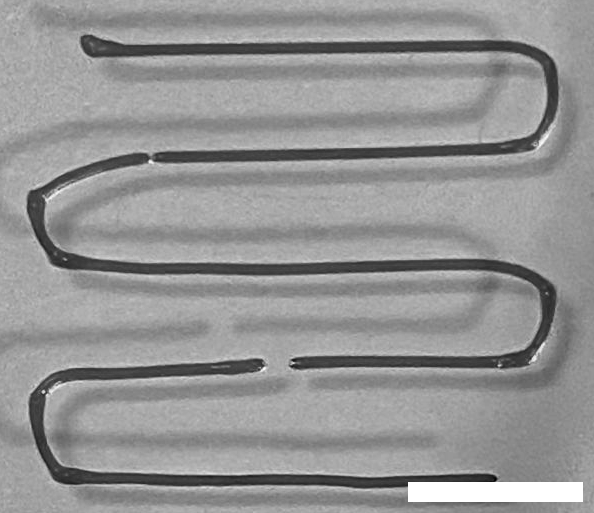

Supplement: S3 Data — (ZIP) [file pone.0312726.s005.zip › Figure 3 data/Filament printing/10mms-1 air. 60kpa 5um scale bar.png]

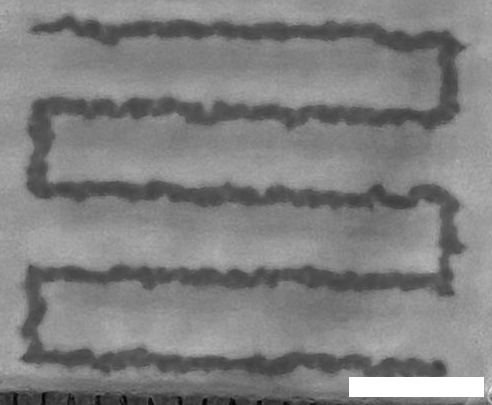

Supplement: S3 Data — (ZIP) [file pone.0312726.s005.zip › Figure 3 data/Filament printing/10mms-1 GG 60kpa 5um scale bar.png]

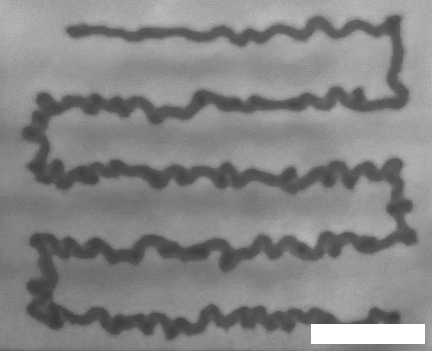

Supplement: S3 Data — (ZIP) [file pone.0312726.s005.zip › Figure 3 data/Filament printing/10mms-1,GG,100kPa, 25G straight 5um scale bar.png]

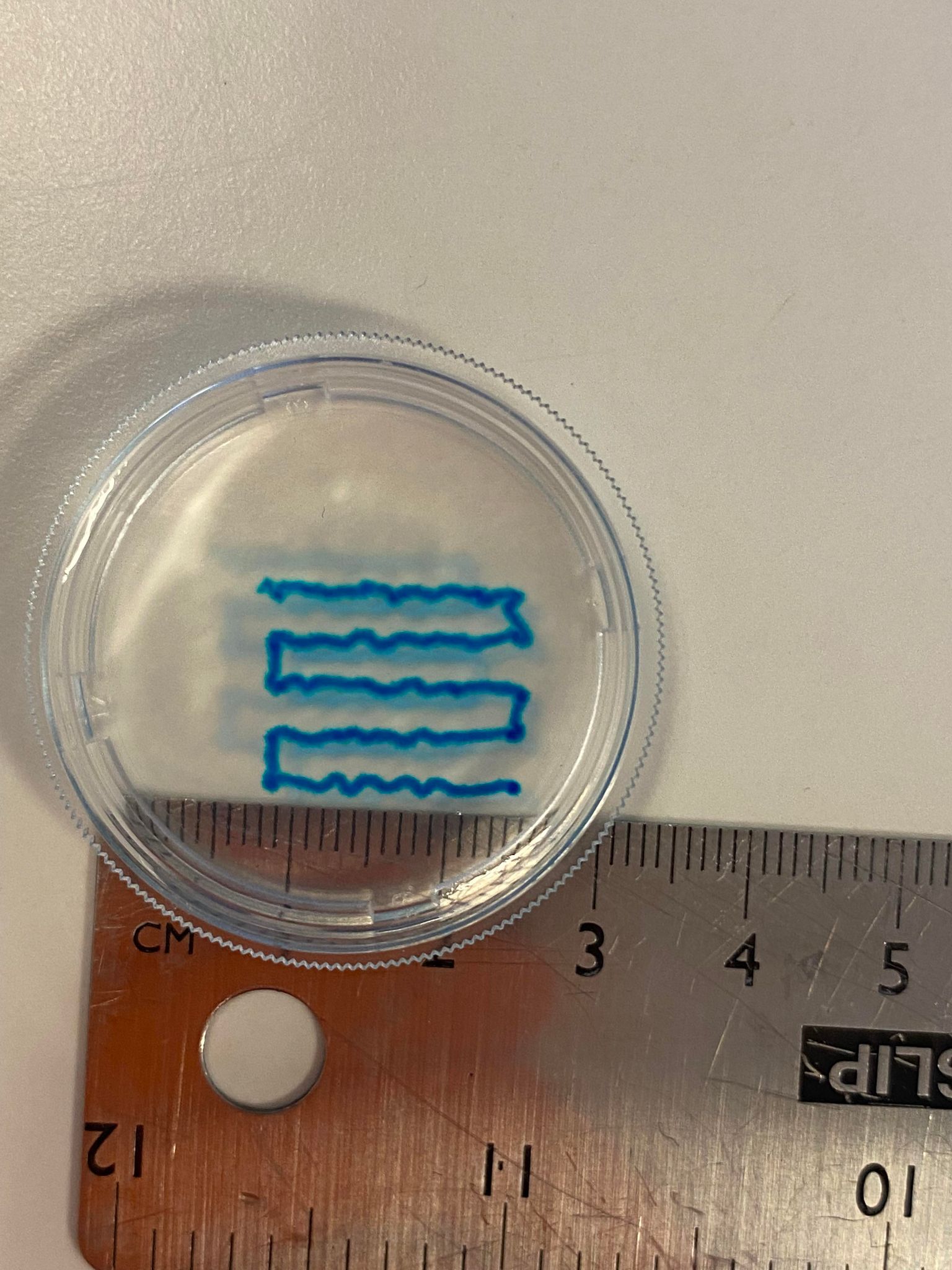

Supplement: S3 Data — (ZIP) [file pone.0312726.s005.zip › Figure 3 data/Filament printing/11.jpg]

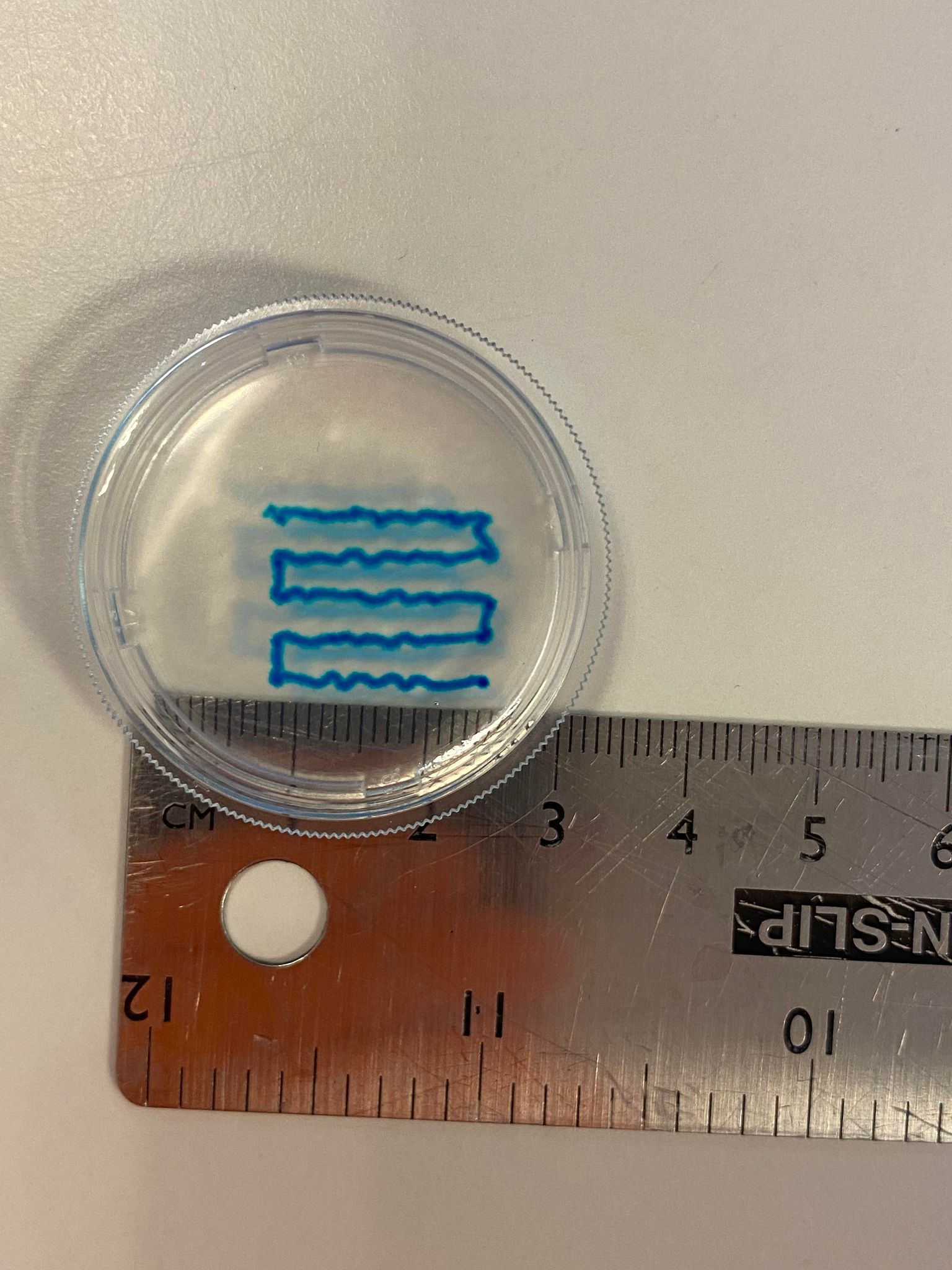

Supplement: S3 Data — (ZIP) [file pone.0312726.s005.zip › Figure 3 data/Filament printing/12.jpg]

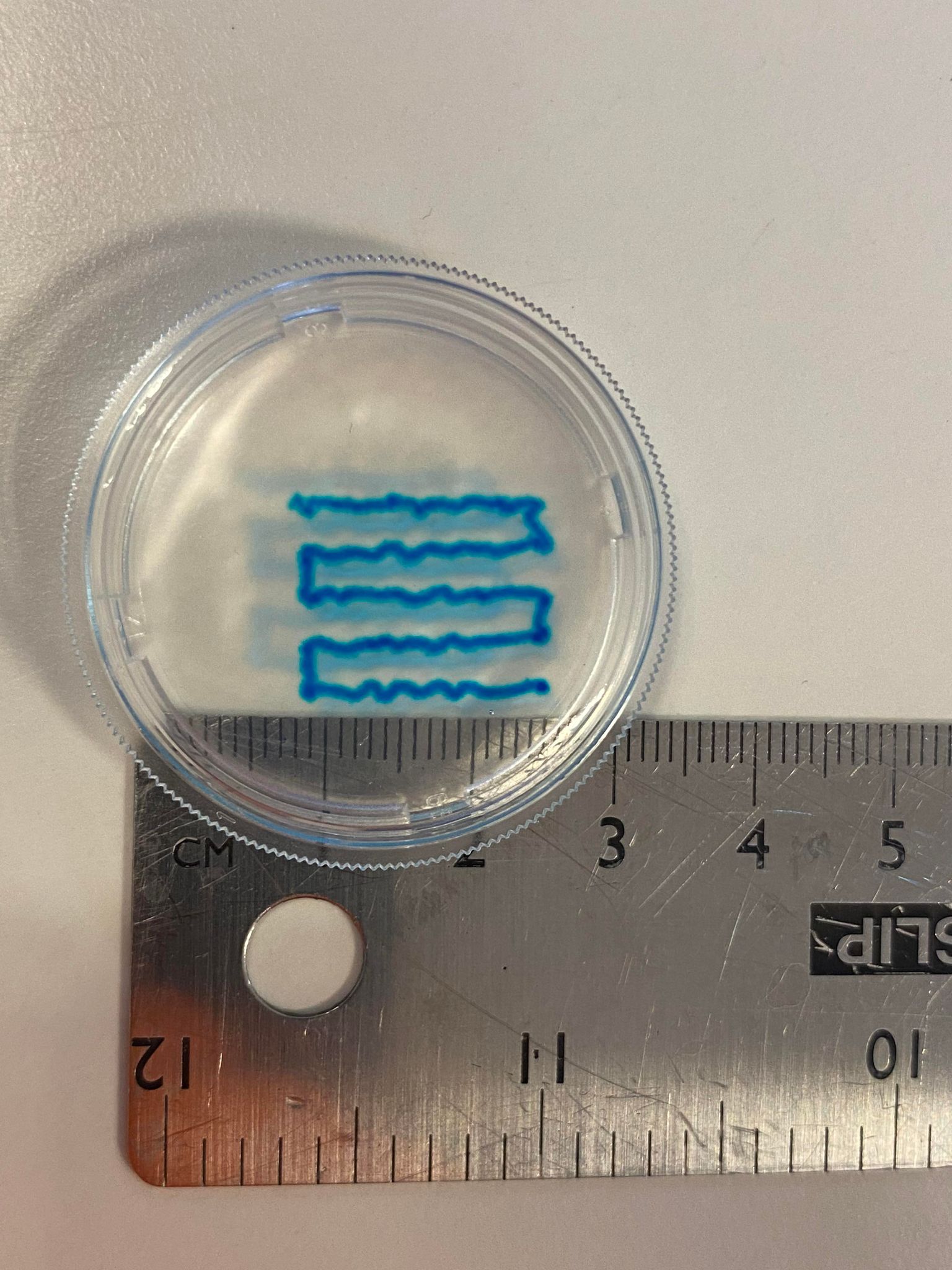

Supplement: S3 Data — (ZIP) [file pone.0312726.s005.zip › Figure 3 data/Filament printing/13.jpg]

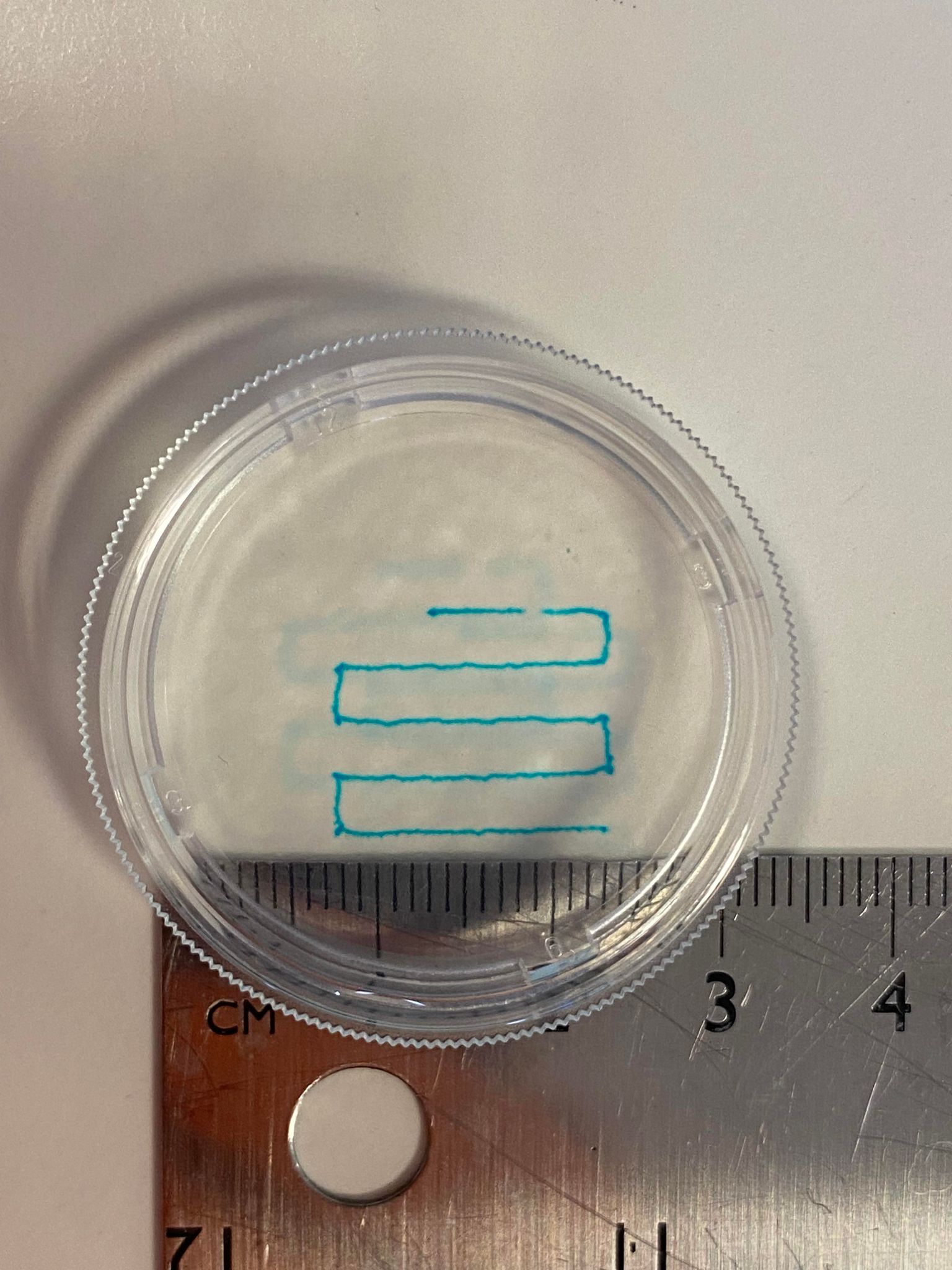

Supplement: S3 Data — (ZIP) [file pone.0312726.s005.zip › Figure 3 data/Filament printing/14.jpg]

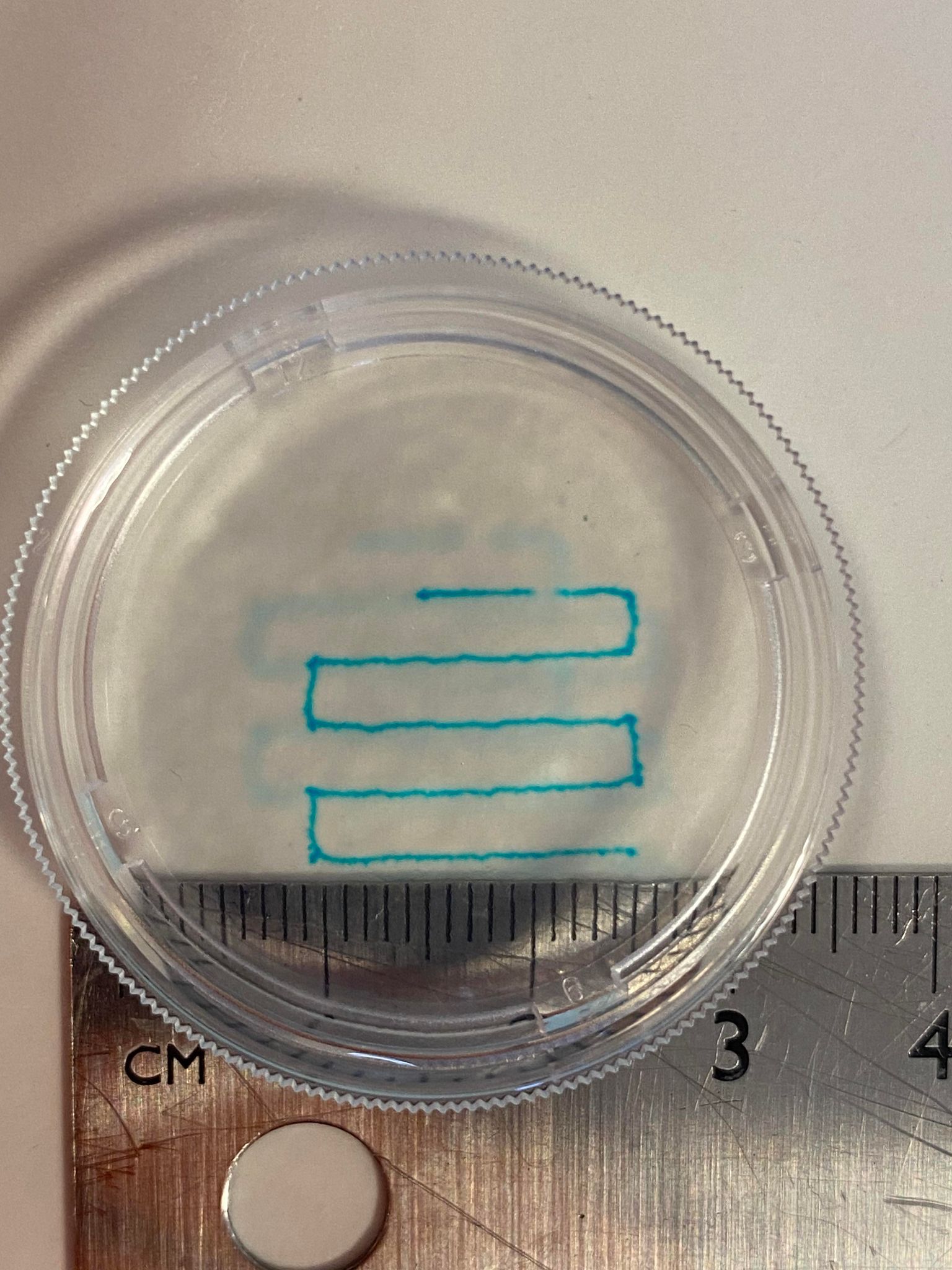

Supplement: S3 Data — (ZIP) [file pone.0312726.s005.zip › Figure 3 data/Filament printing/15.jpg]

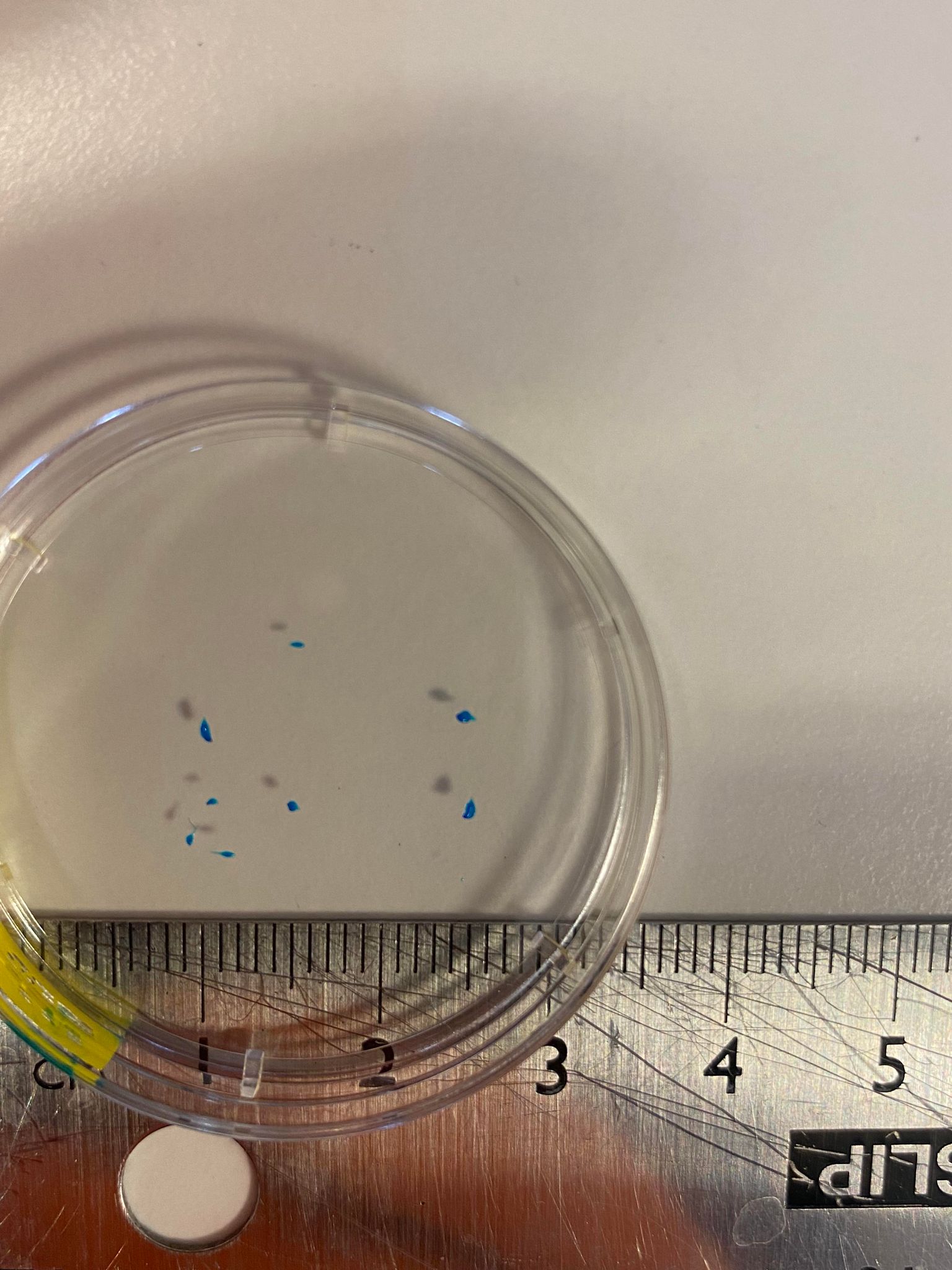

Supplement: S3 Data — (ZIP) [file pone.0312726.s005.zip › Figure 3 data/Filament printing/16.jpg]

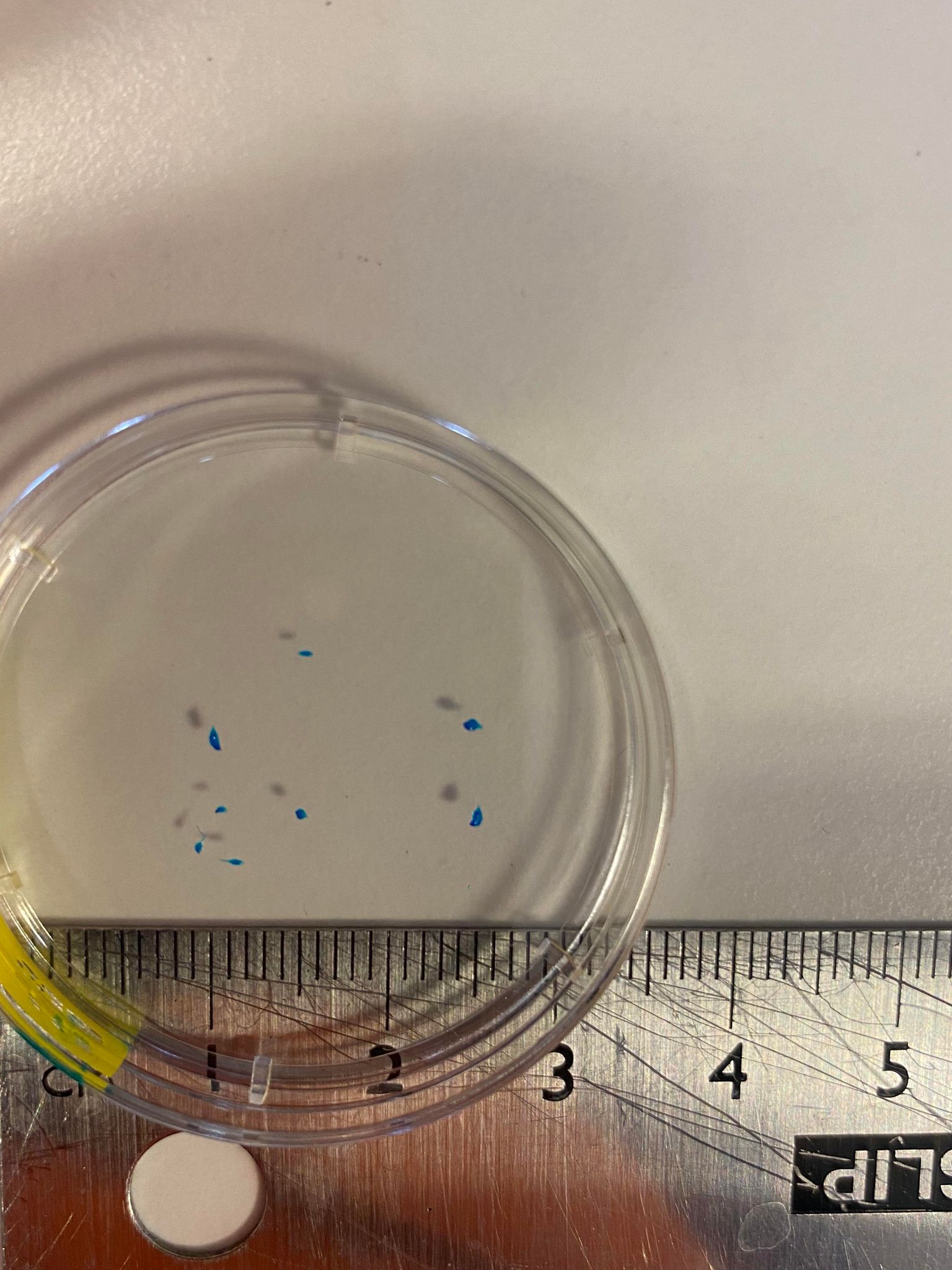

Supplement: S3 Data — (ZIP) [file pone.0312726.s005.zip › Figure 3 data/Filament printing/17.jpg]

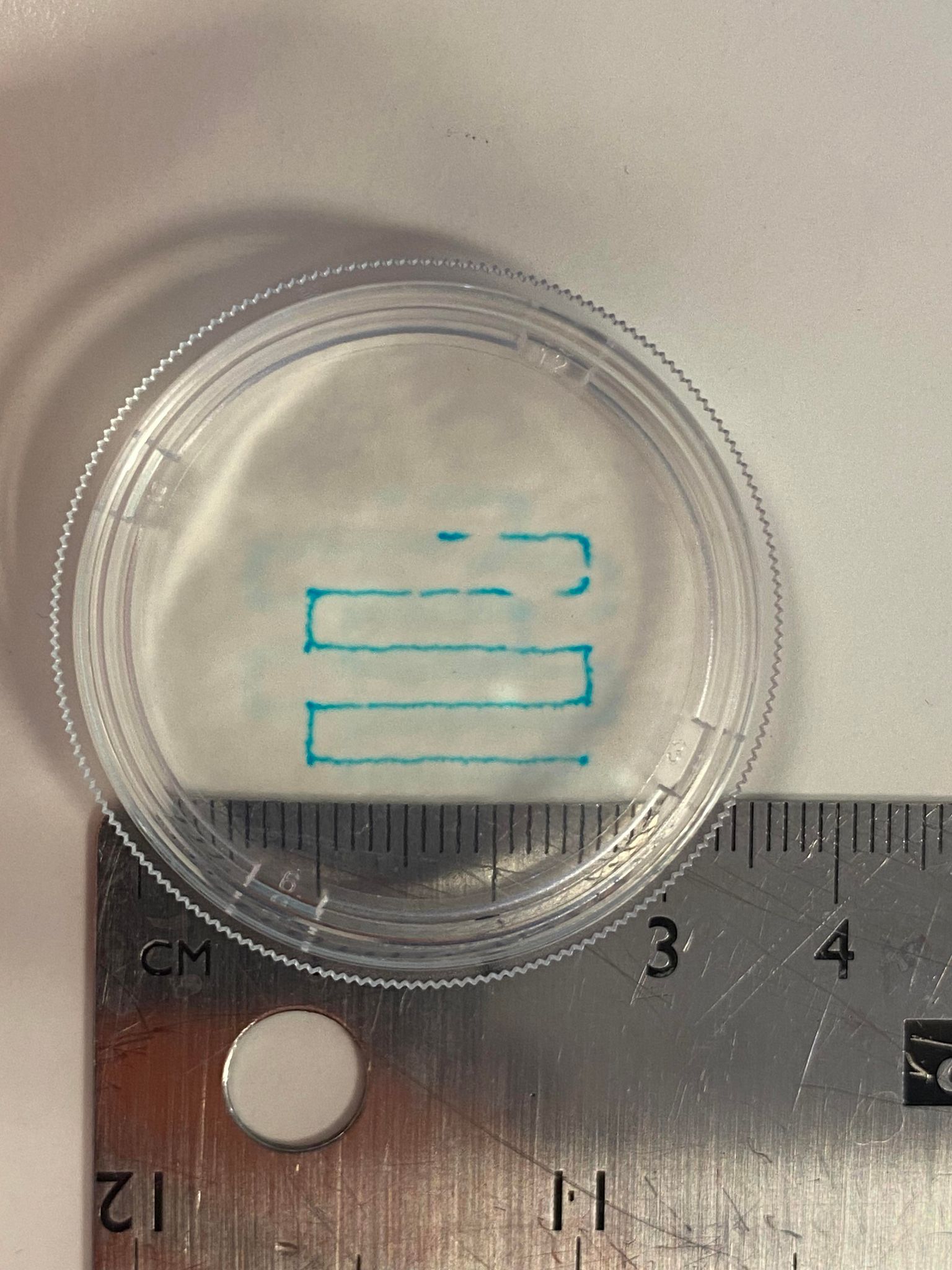

Supplement: S3 Data — (ZIP) [file pone.0312726.s005.zip › Figure 3 data/Filament printing/18.jpg]

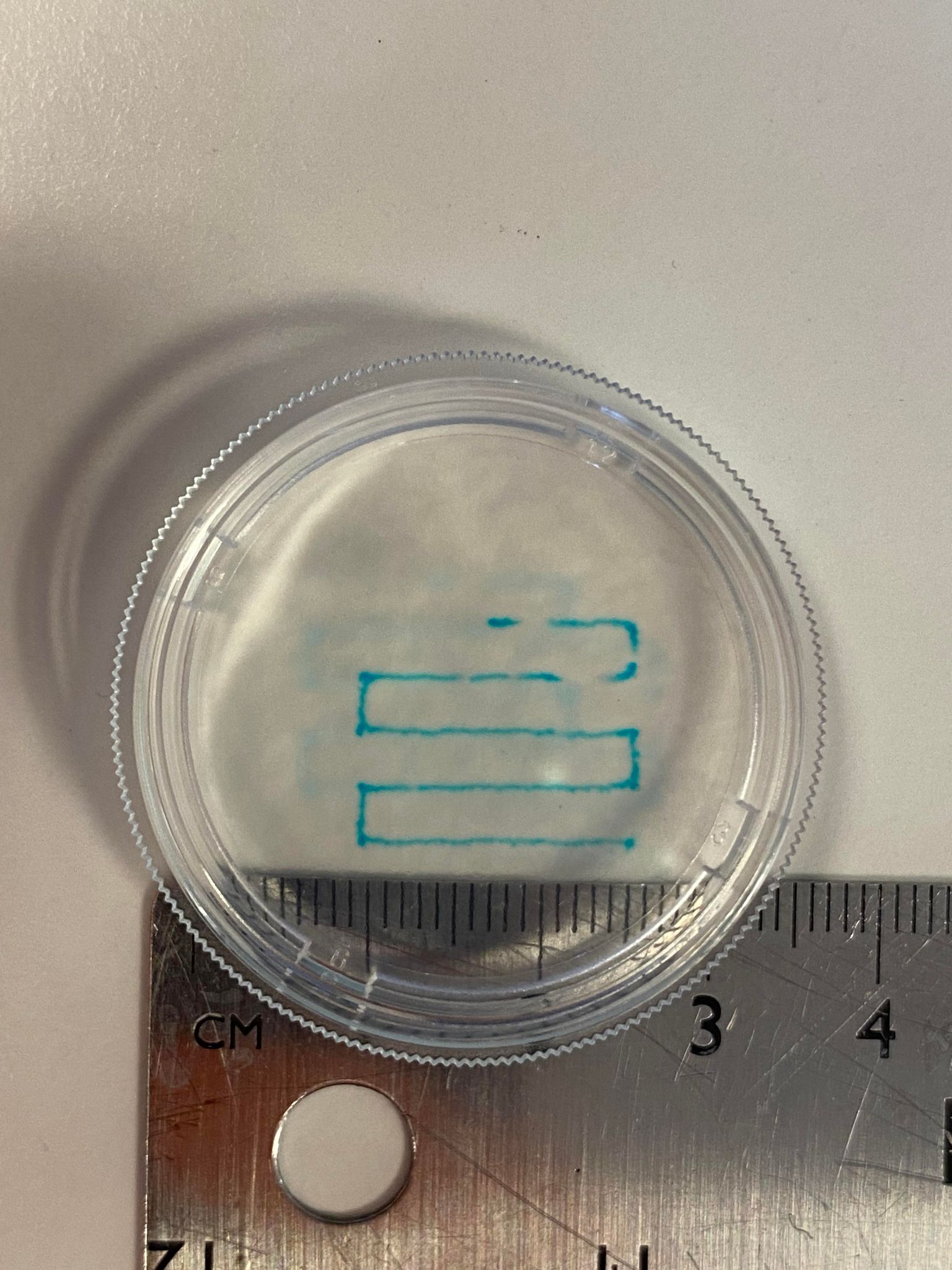

Supplement: S3 Data — (ZIP) [file pone.0312726.s005.zip › Figure 3 data/Filament printing/19.jpg]

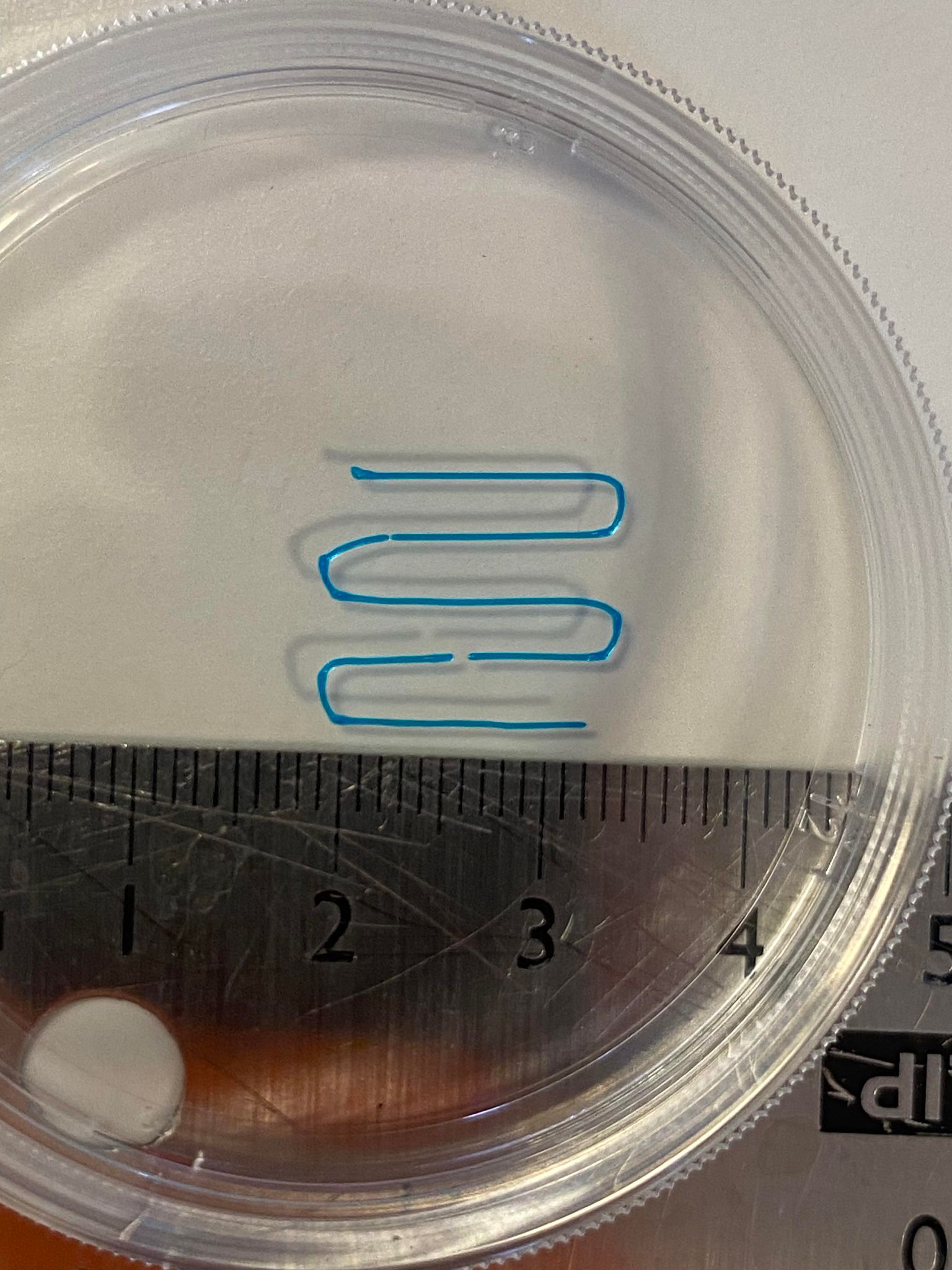

Supplement: S3 Data — (ZIP) [file pone.0312726.s005.zip › Figure 3 data/Filament printing/2.jpg]

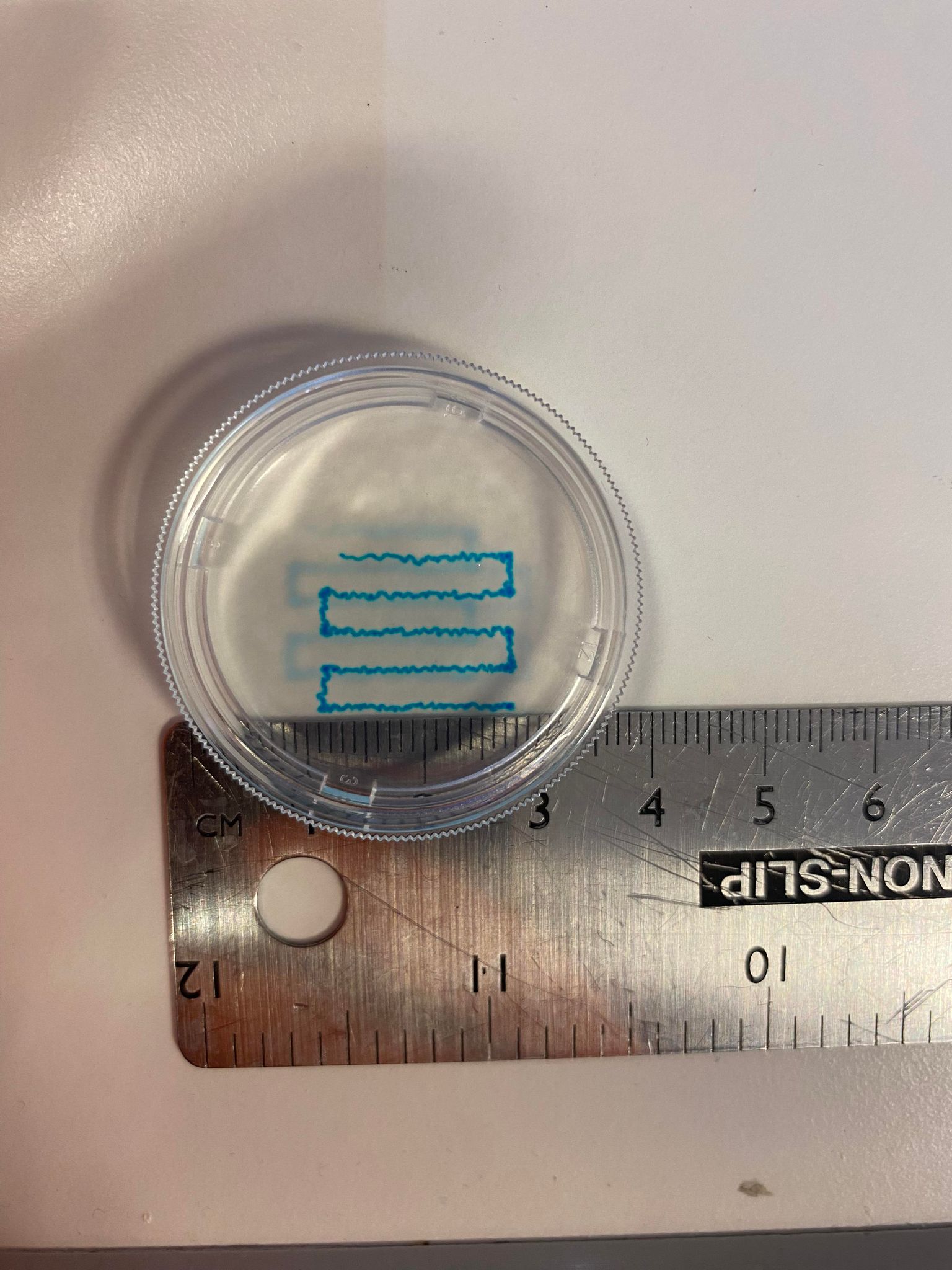

Supplement: S3 Data — (ZIP) [file pone.0312726.s005.zip › Figure 3 data/Filament printing/20.jpg]

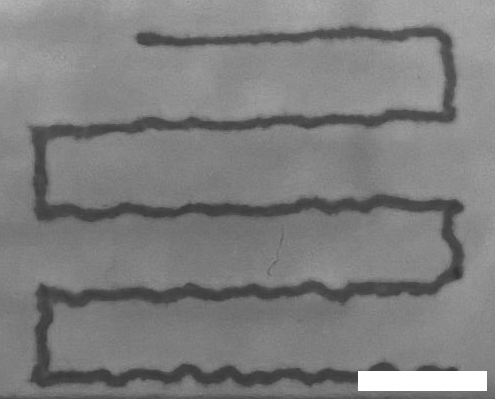

Supplement: S3 Data — (ZIP) [file pone.0312726.s005.zip › Figure 3 data/Filament printing/20mms-1,GG,100kPa, 25G straight 5um scale bar.png]

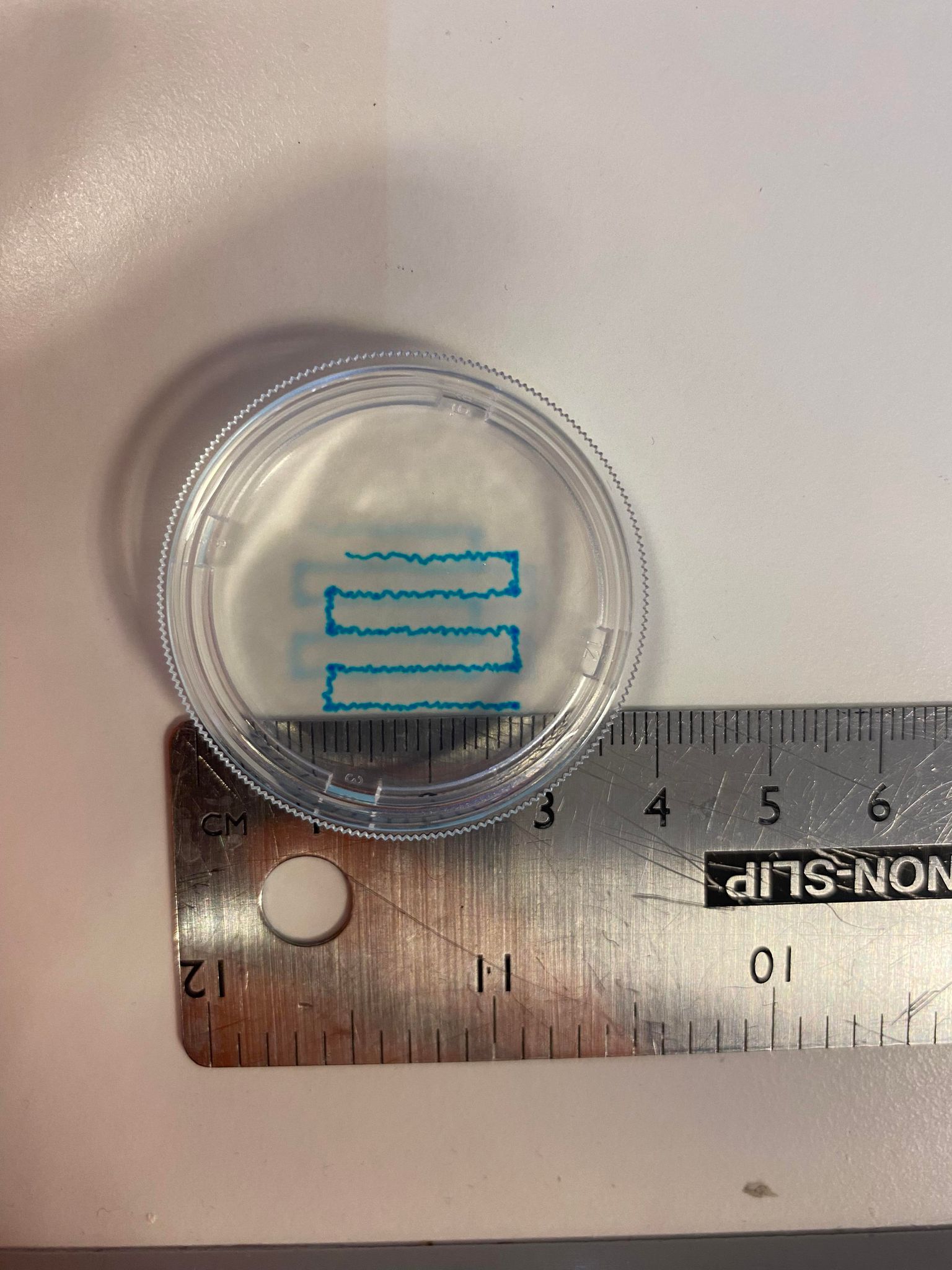

Supplement: S3 Data — (ZIP) [file pone.0312726.s005.zip › Figure 3 data/Filament printing/21.jpg]

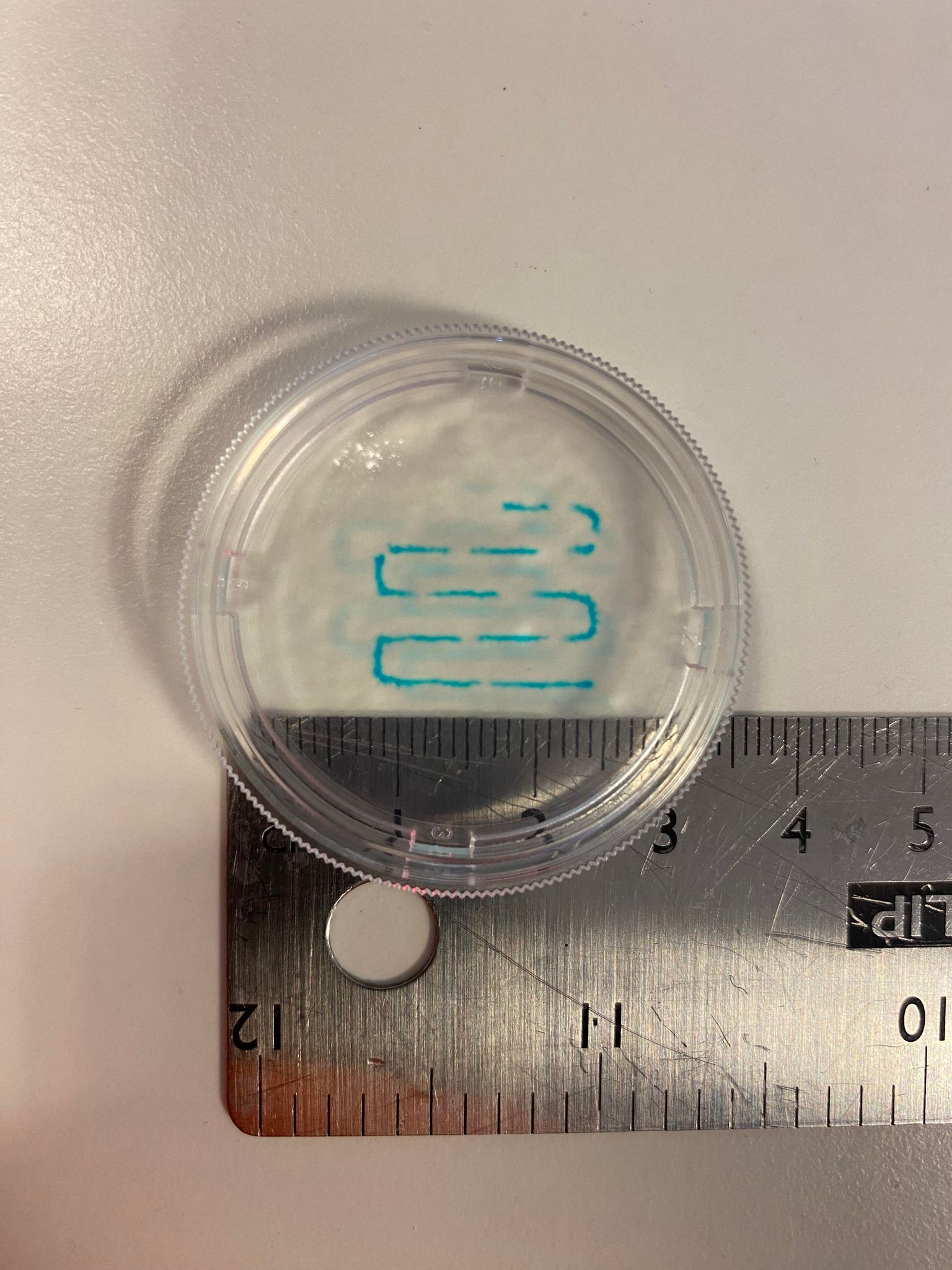

Supplement: S3 Data — (ZIP) [file pone.0312726.s005.zip › Figure 3 data/Filament printing/22.jpg]

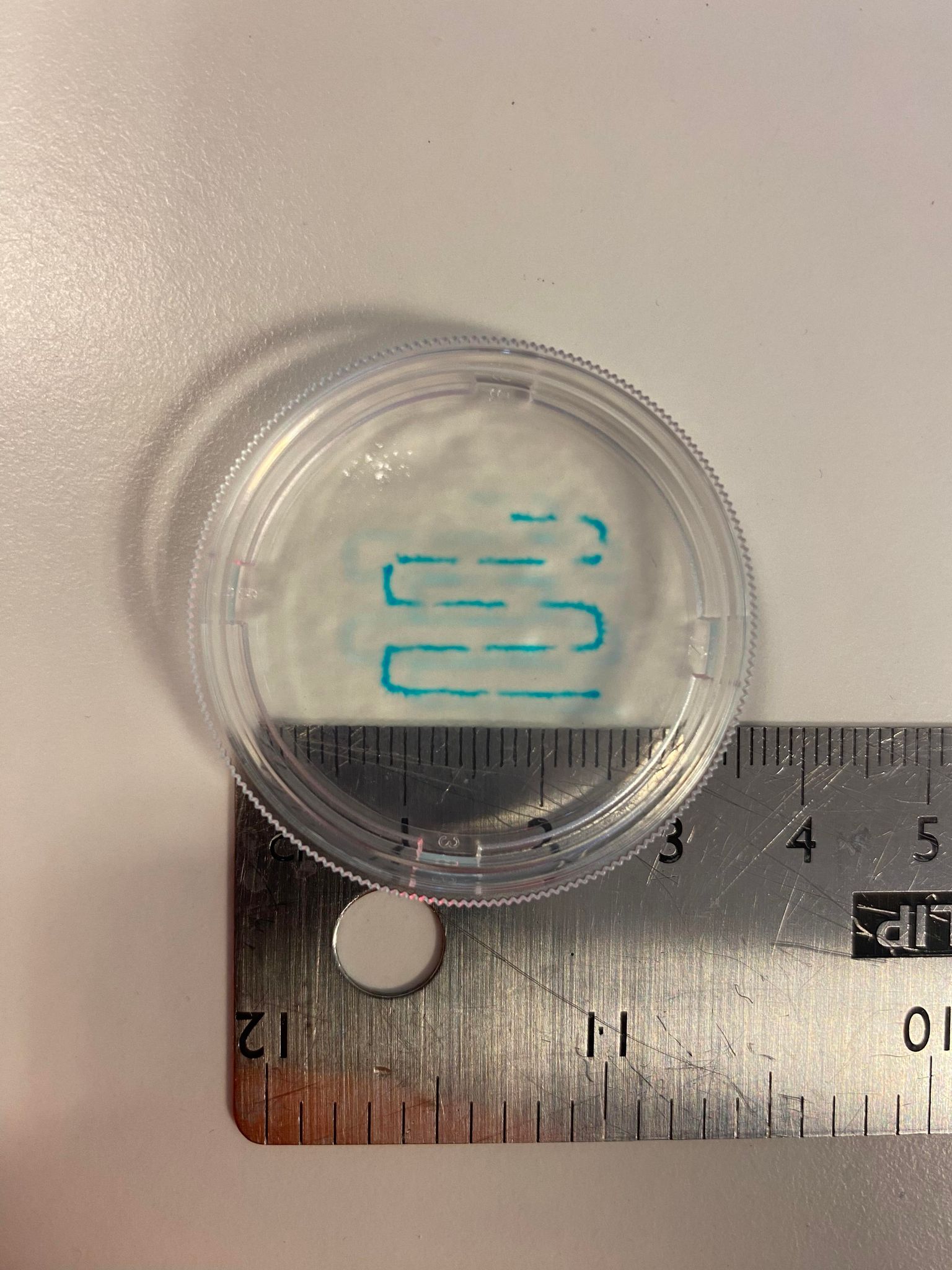

Supplement: S3 Data — (ZIP) [file pone.0312726.s005.zip › Figure 3 data/Filament printing/23.jpg]

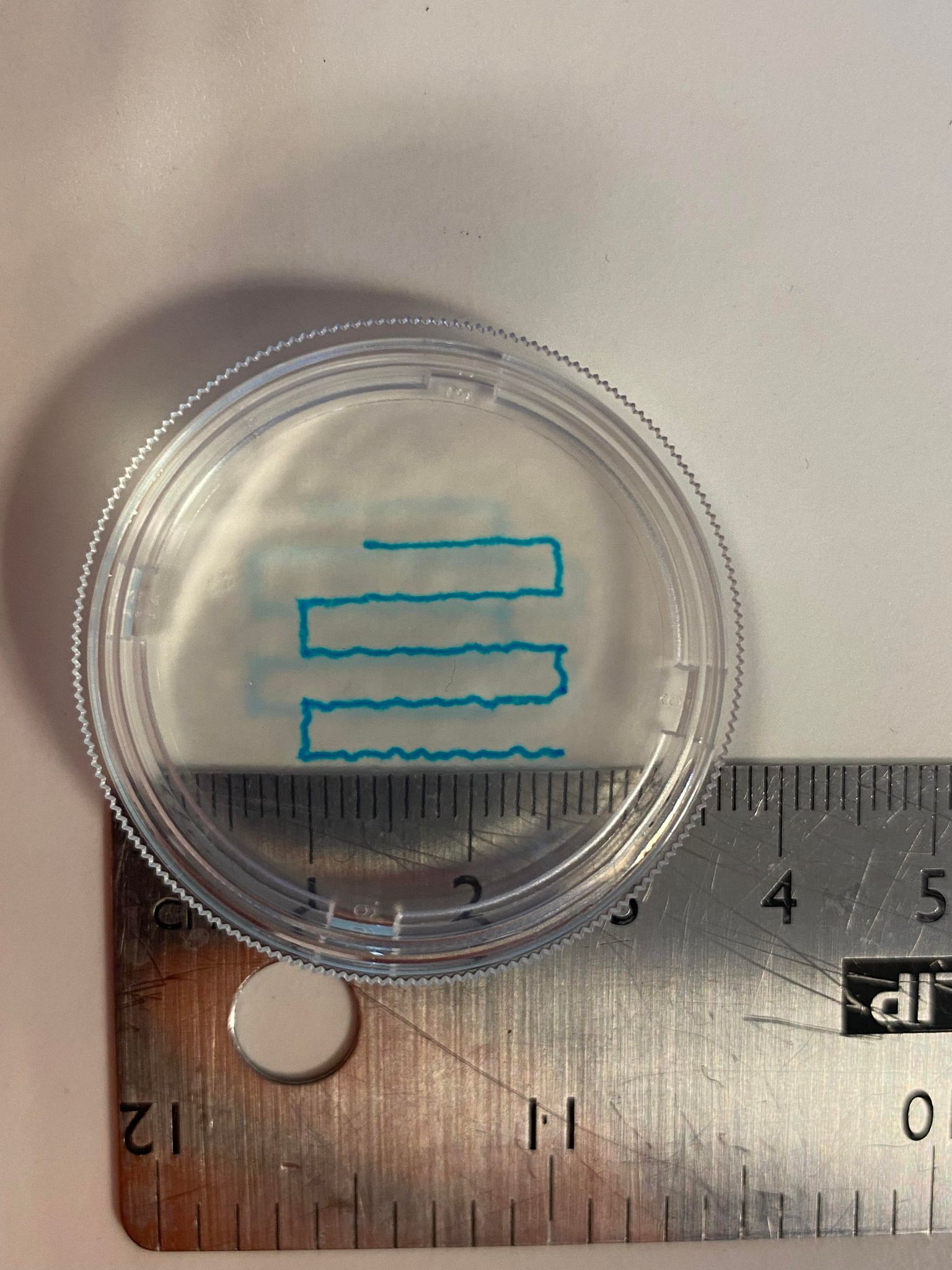

Supplement: S3 Data — (ZIP) [file pone.0312726.s005.zip › Figure 3 data/Filament printing/24.jpg]

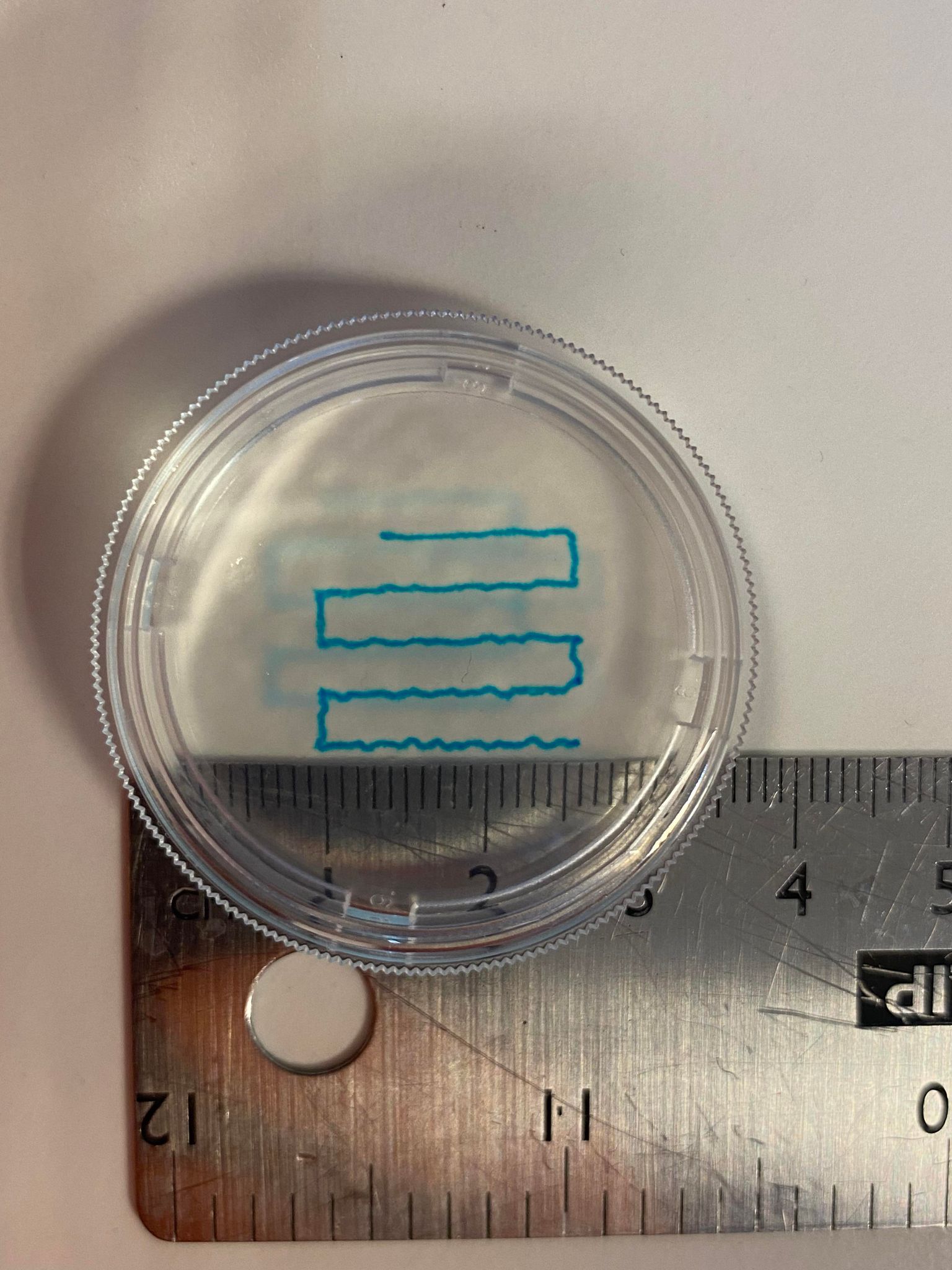

Supplement: S3 Data — (ZIP) [file pone.0312726.s005.zip › Figure 3 data/Filament printing/25.jpg]

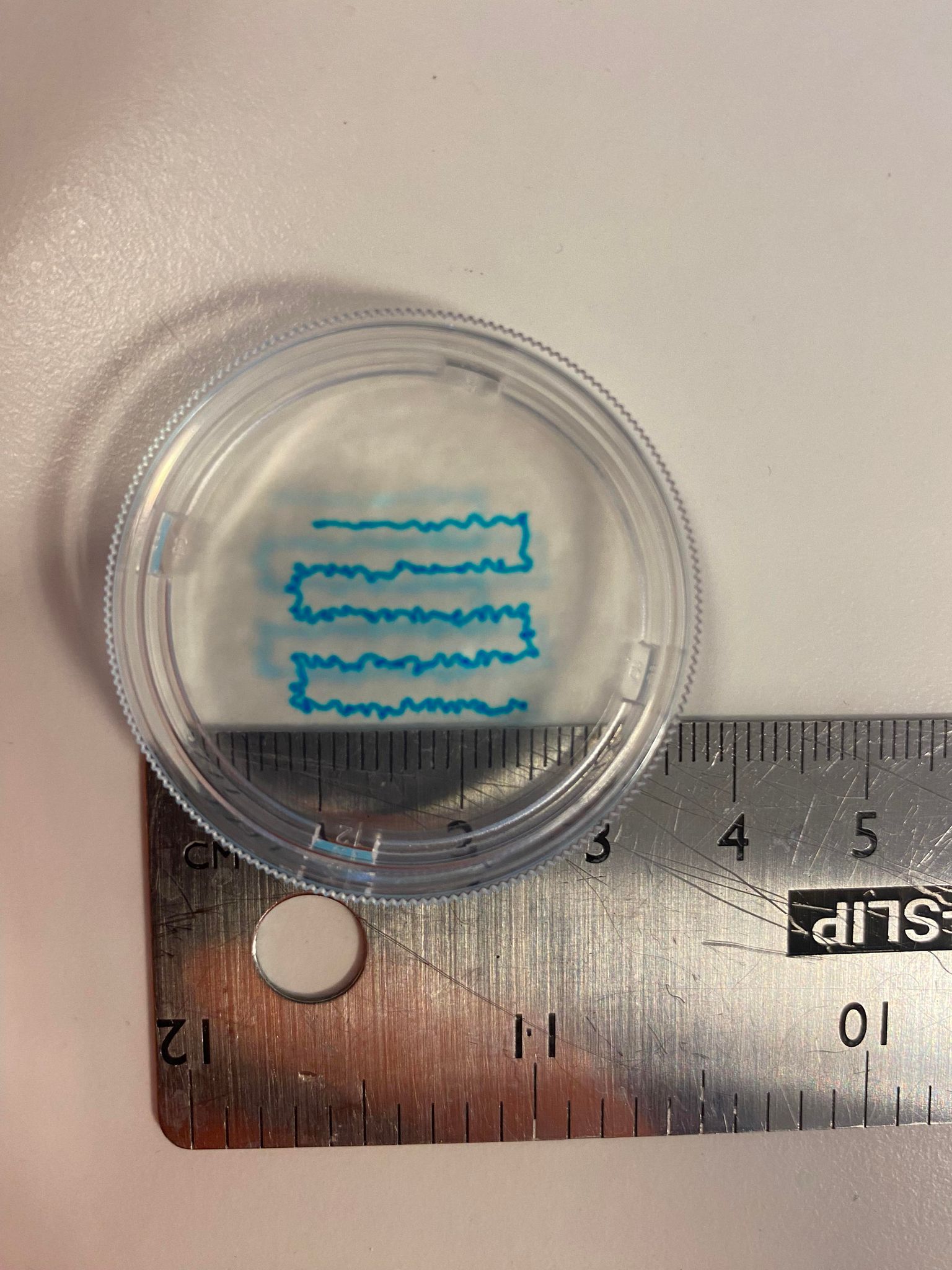

Supplement: S3 Data — (ZIP) [file pone.0312726.s005.zip › Figure 3 data/Filament printing/26.jpg]

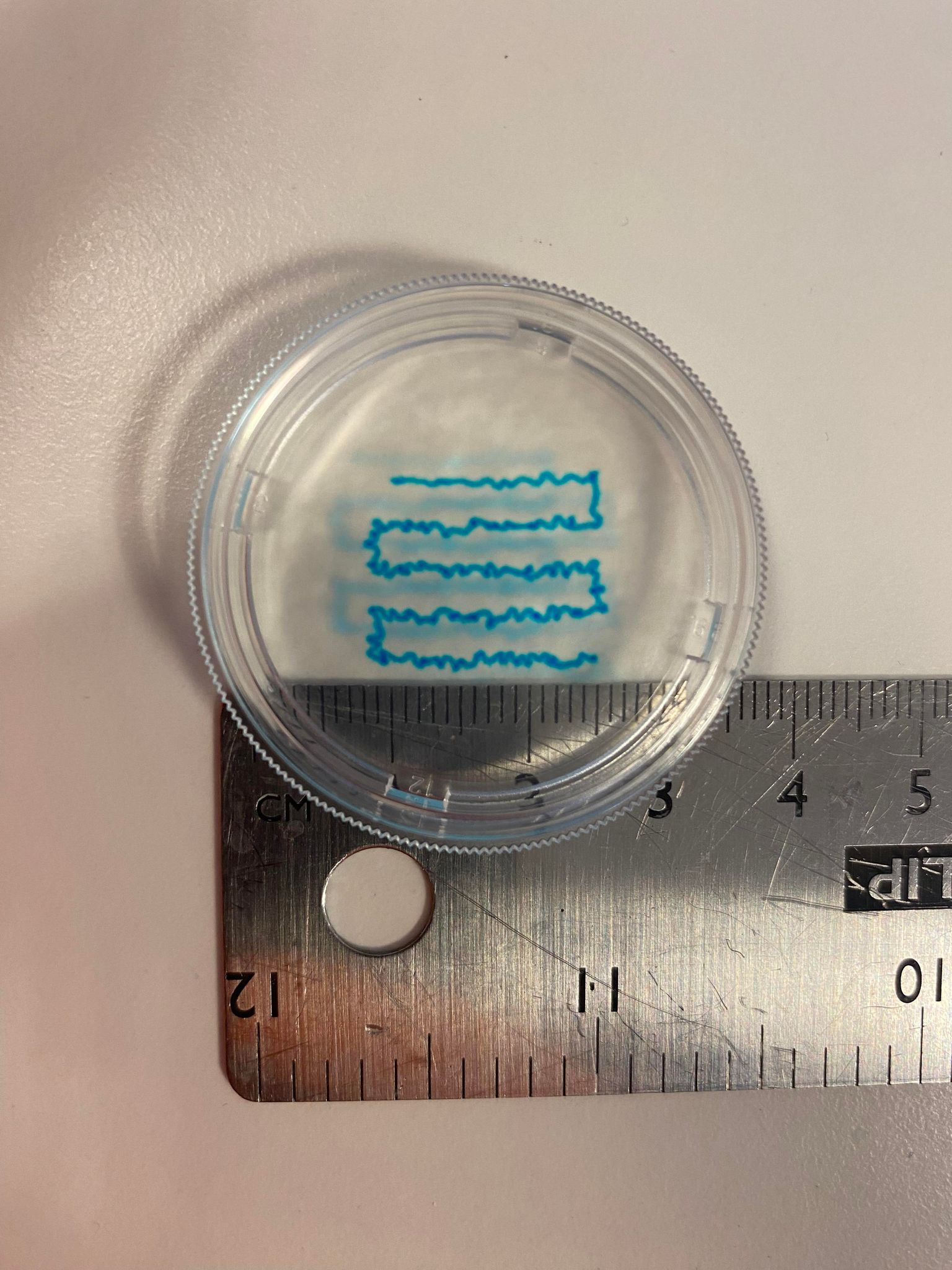

Supplement: S3 Data — (ZIP) [file pone.0312726.s005.zip › Figure 3 data/Filament printing/27.jpg]

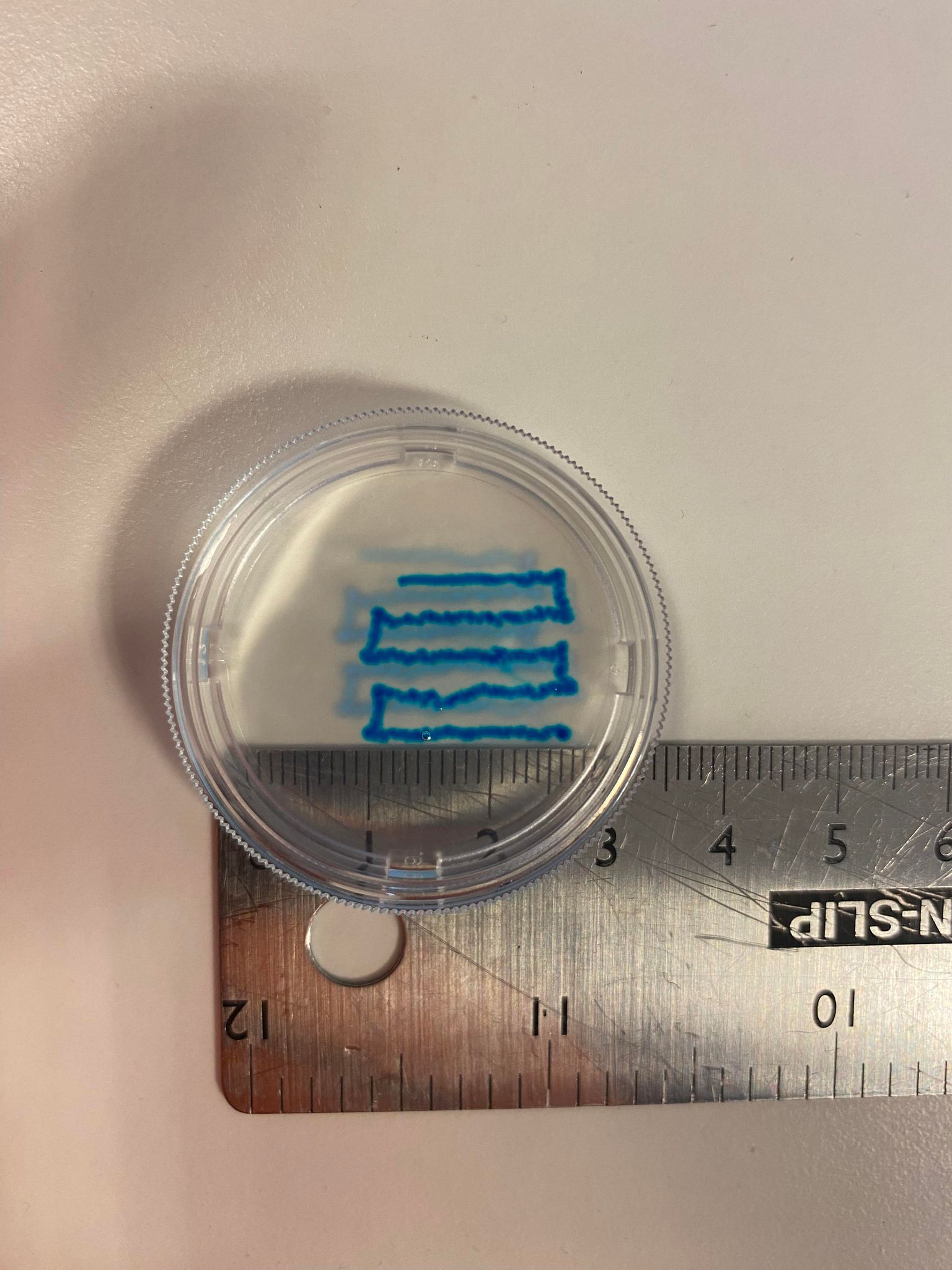

Supplement: S3 Data — (ZIP) [file pone.0312726.s005.zip › Figure 3 data/Filament printing/28.jpg]

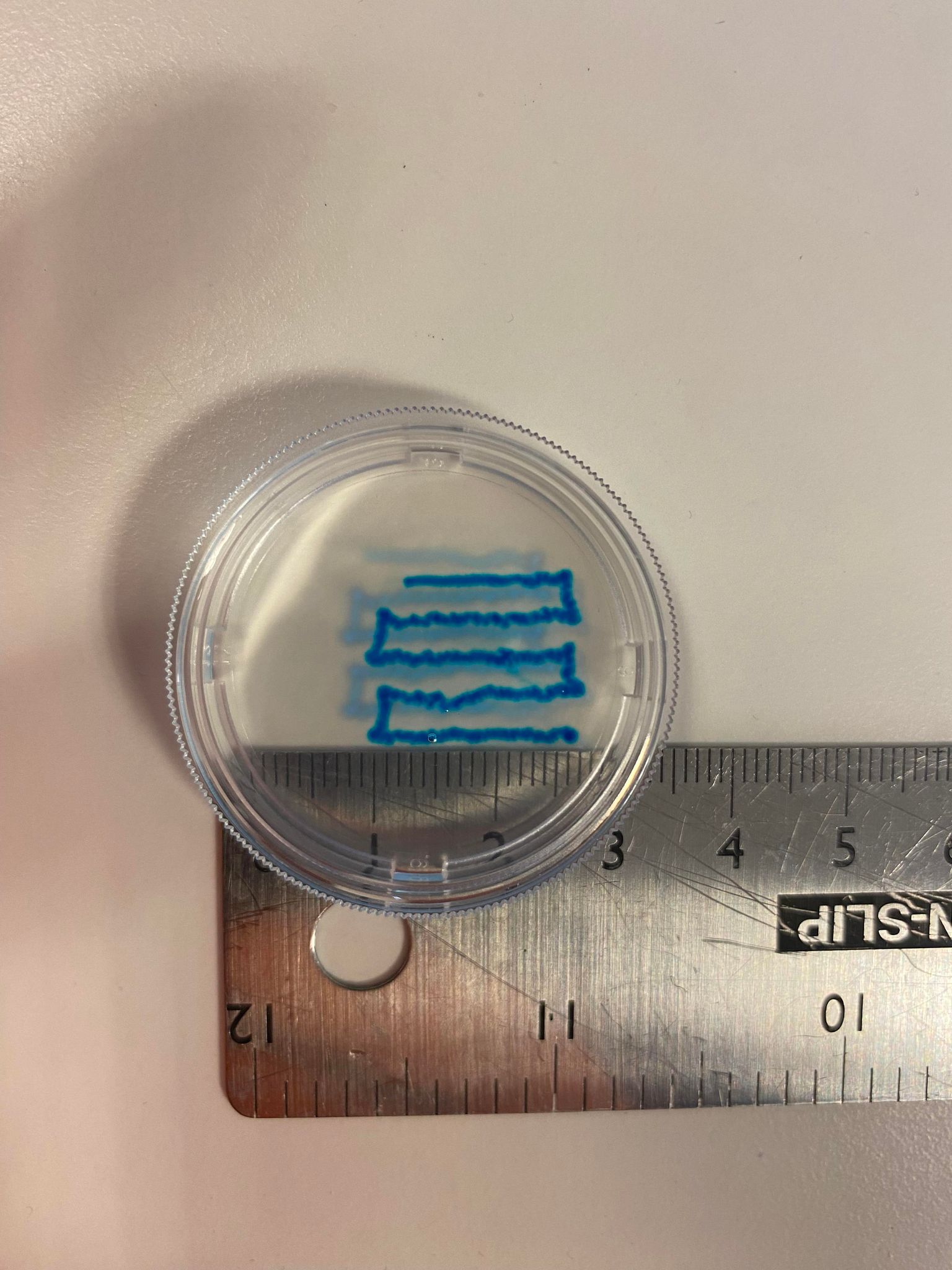

Supplement: S3 Data — (ZIP) [file pone.0312726.s005.zip › Figure 3 data/Filament printing/29.jpg]

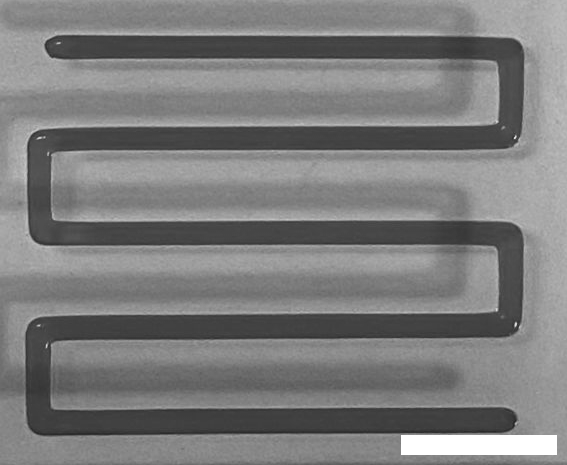

Supplement: S3 Data — (ZIP) [file pone.0312726.s005.zip › Figure 3 data/Filament printing/2mms-1 air. 60kpa 5um scale bar.png]

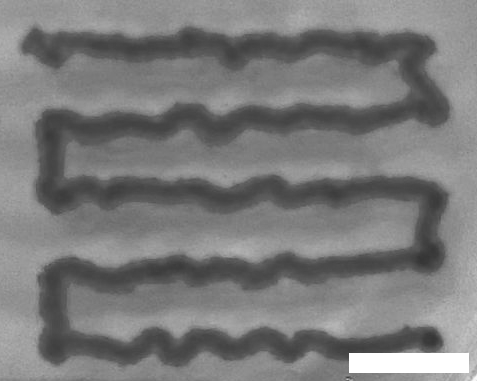

Supplement: S3 Data — (ZIP) [file pone.0312726.s005.zip › Figure 3 data/Filament printing/2mms-1 GG 60kpa 5um scale bar.png]

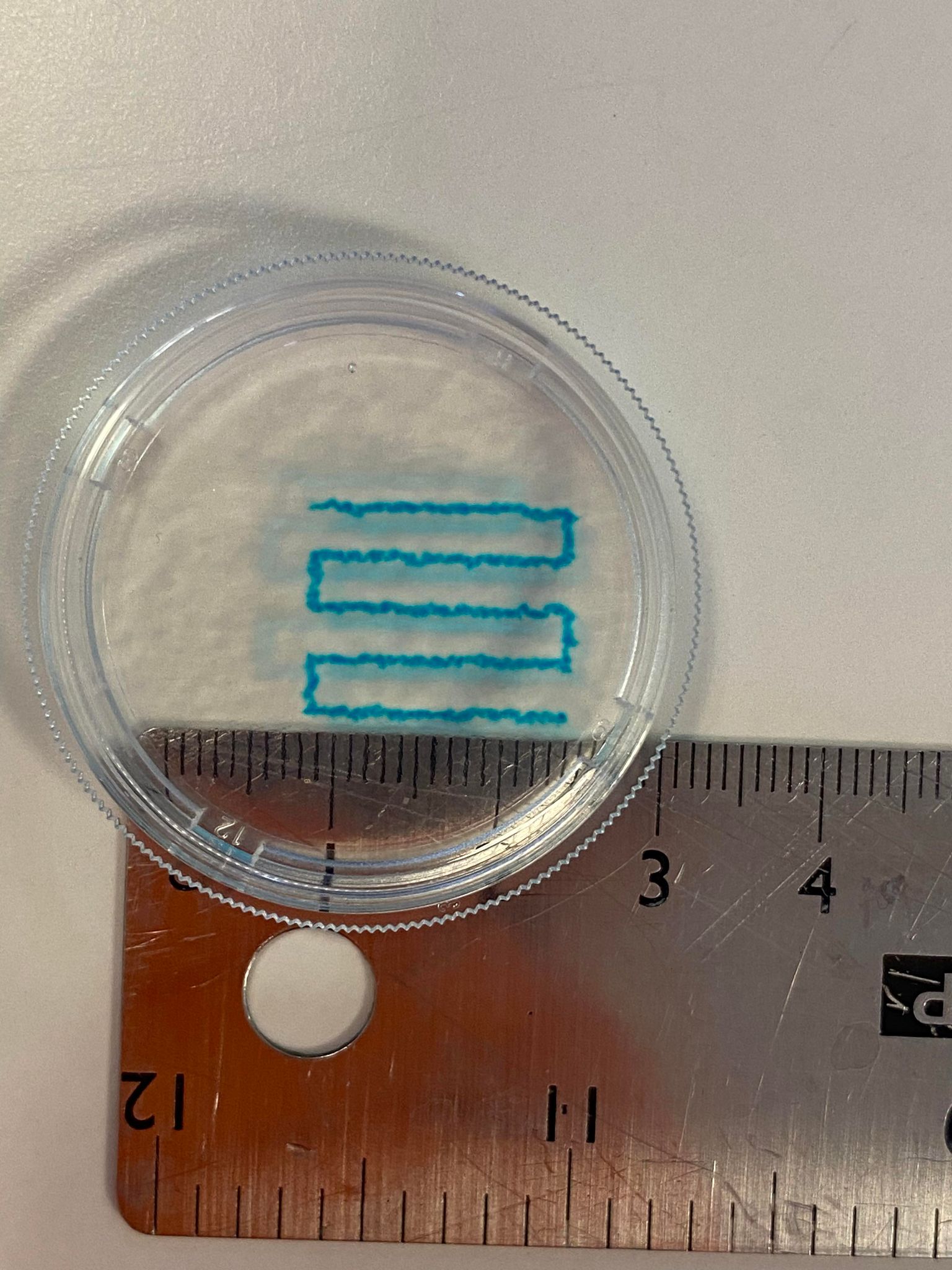

Supplement: S3 Data — (ZIP) [file pone.0312726.s005.zip › Figure 3 data/Filament printing/3.jpg]

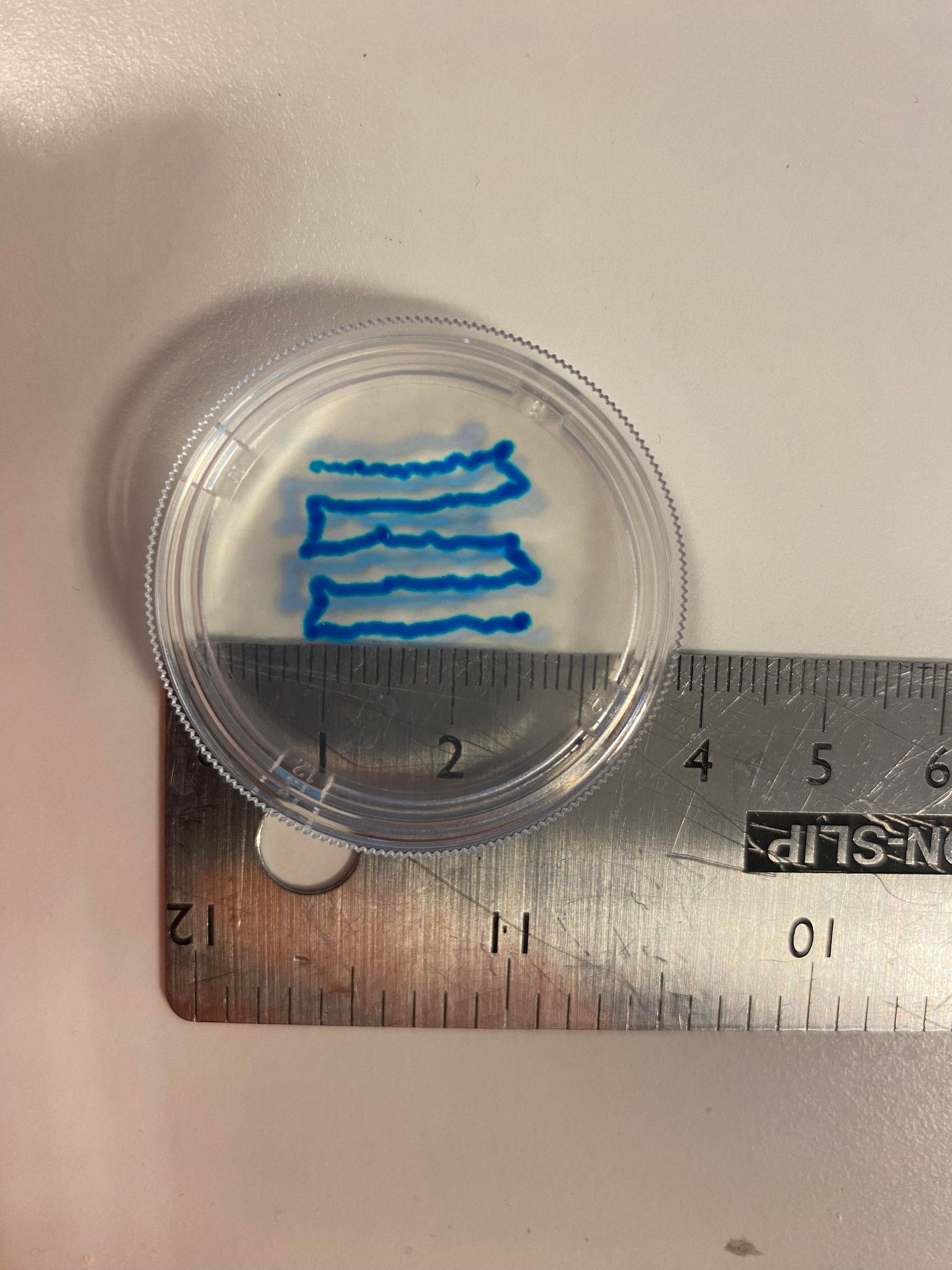

Supplement: S3 Data — (ZIP) [file pone.0312726.s005.zip › Figure 3 data/Filament printing/30.jpg]

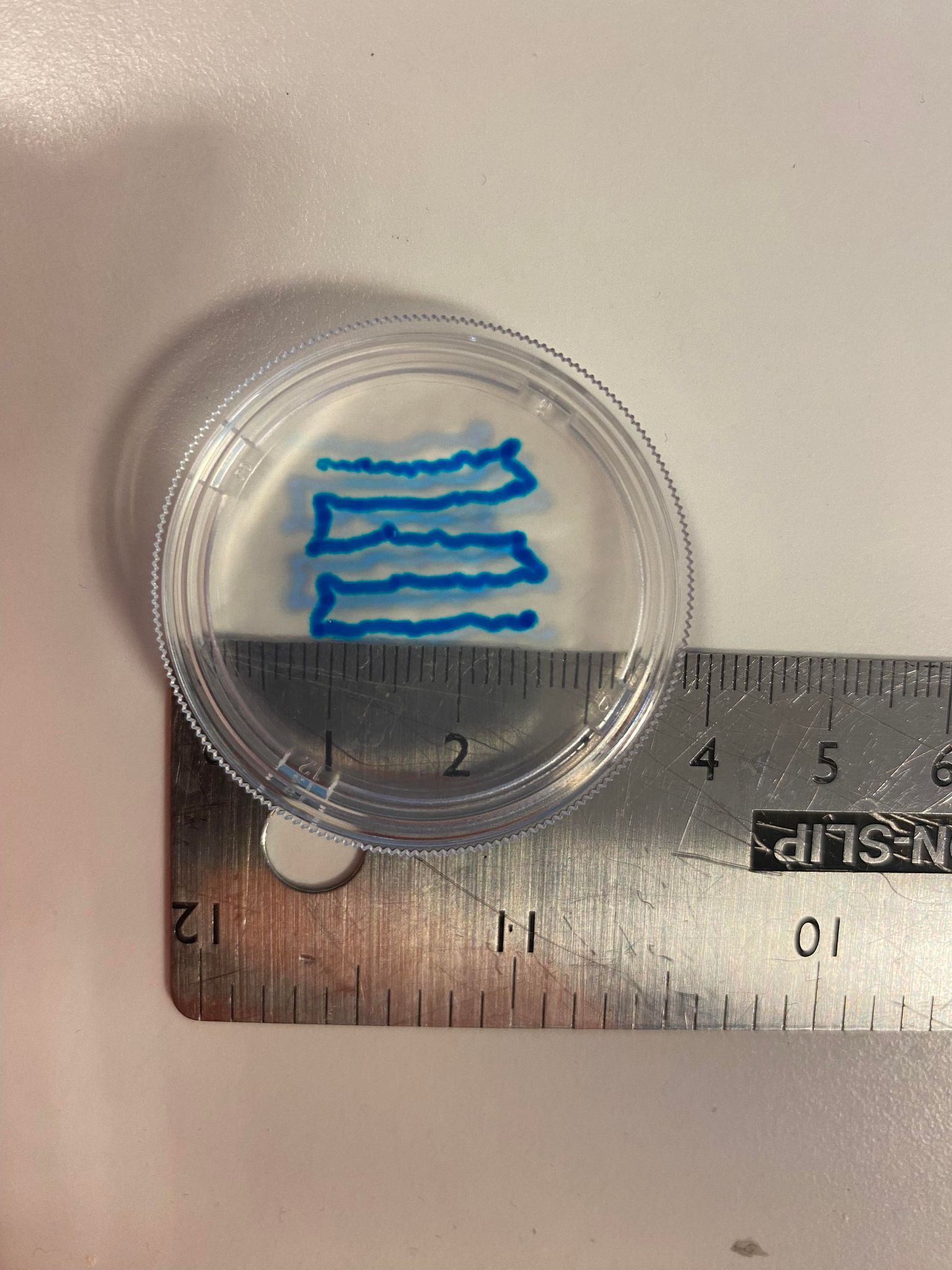

Supplement: S3 Data — (ZIP) [file pone.0312726.s005.zip › Figure 3 data/Filament printing/31.jpg]

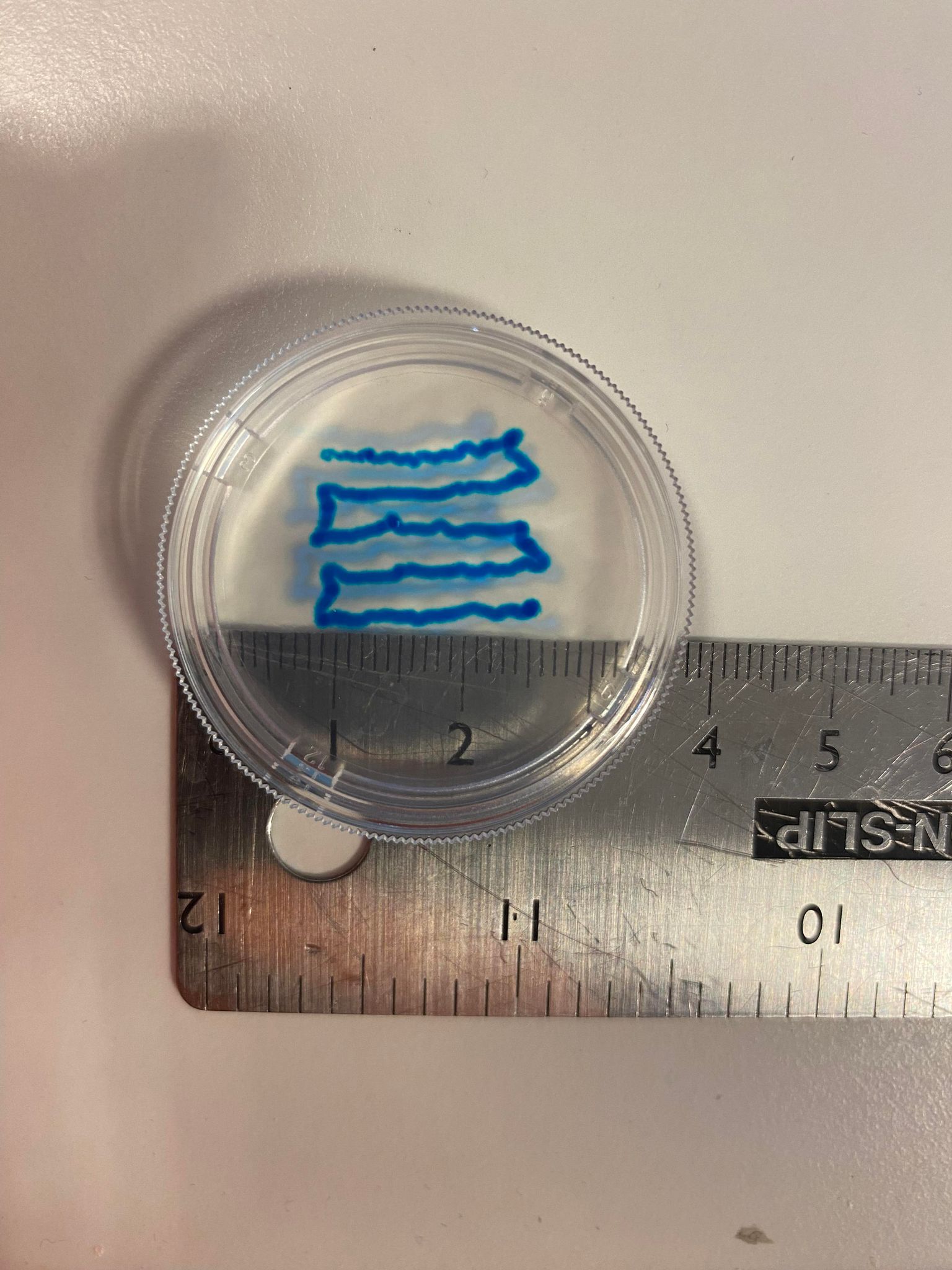

Supplement: S3 Data — (ZIP) [file pone.0312726.s005.zip › Figure 3 data/Filament printing/32.jpg]

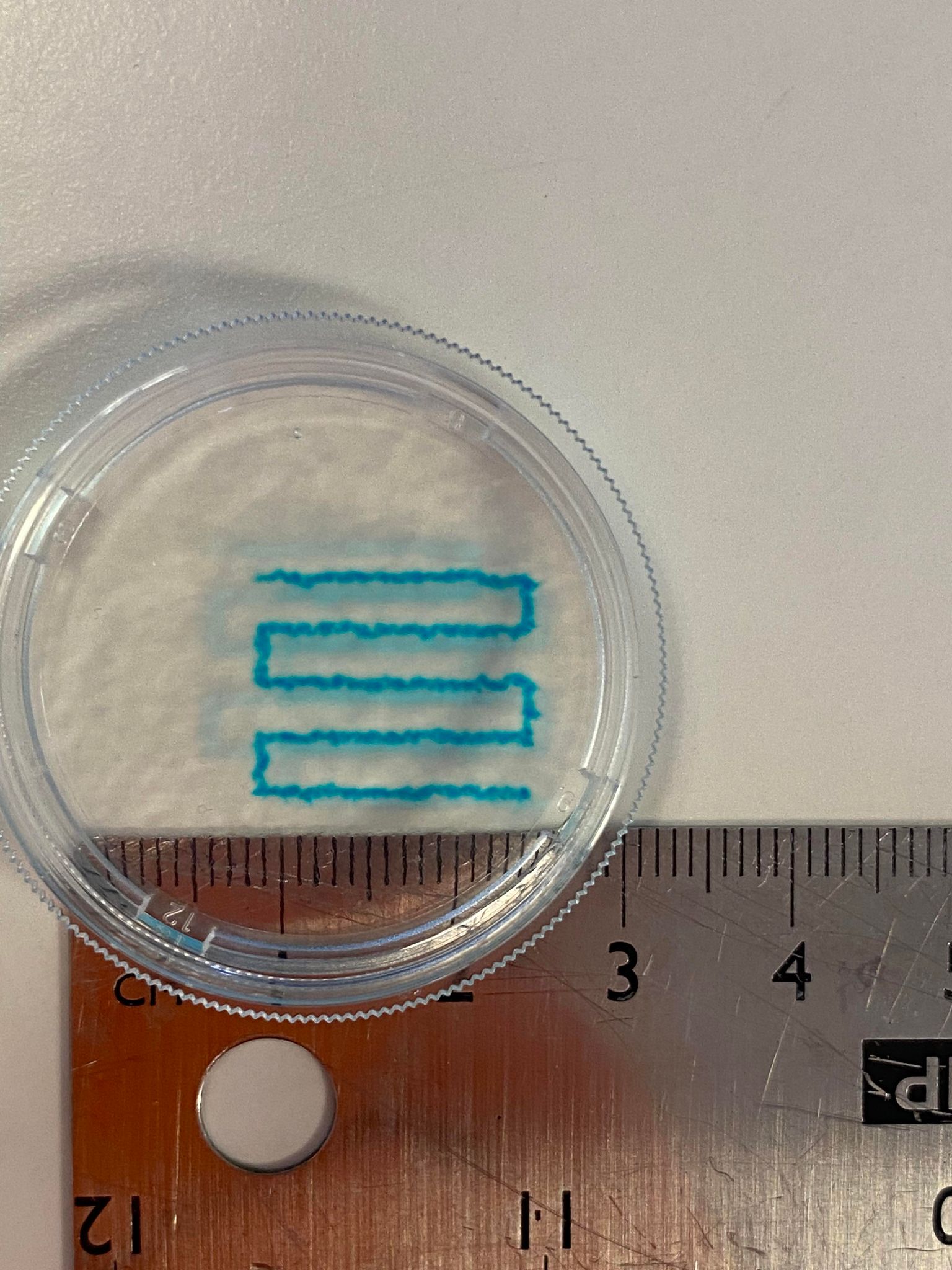

Supplement: S3 Data — (ZIP) [file pone.0312726.s005.zip › Figure 3 data/Filament printing/4.jpg]

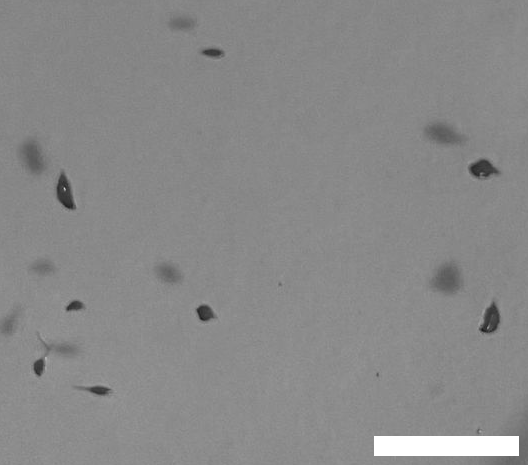

Supplement: S3 Data — (ZIP) [file pone.0312726.s005.zip › Figure 3 data/Filament printing/40mms-1 air. 60kpa 5um scale bar.png]

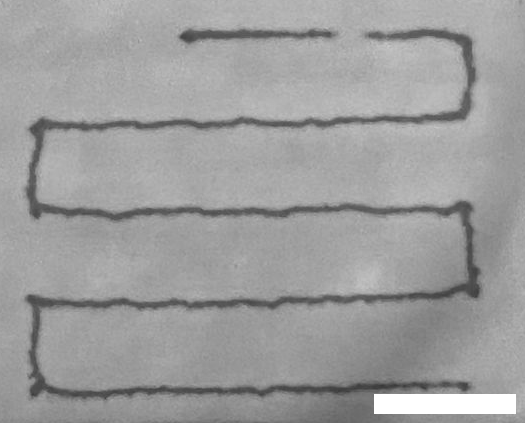

Supplement: S3 Data — (ZIP) [file pone.0312726.s005.zip › Figure 3 data/Filament printing/40mms-1 GG 60kpa 5um scale bar.png]

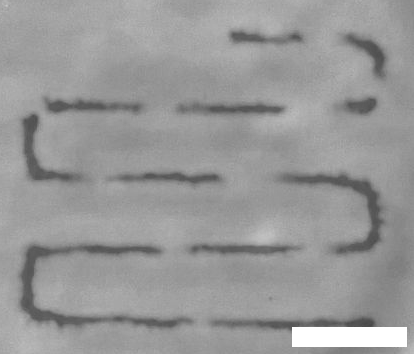

Supplement: S3 Data — (ZIP) [file pone.0312726.s005.zip › Figure 3 data/Filament printing/40mms-1,GG,100kPa, 25G straight 5um scale bar.png]

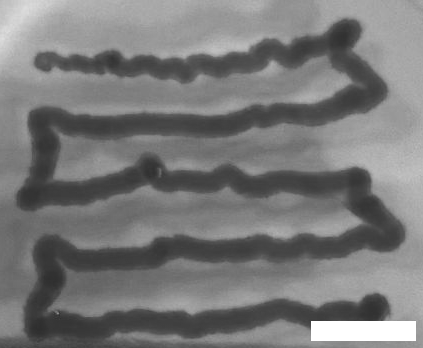

Supplement: S3 Data — (ZIP) [file pone.0312726.s005.zip › Figure 3 data/Filament printing/40mms-1,GG,100kPa, 25G tapered, 5um scale bar.png]

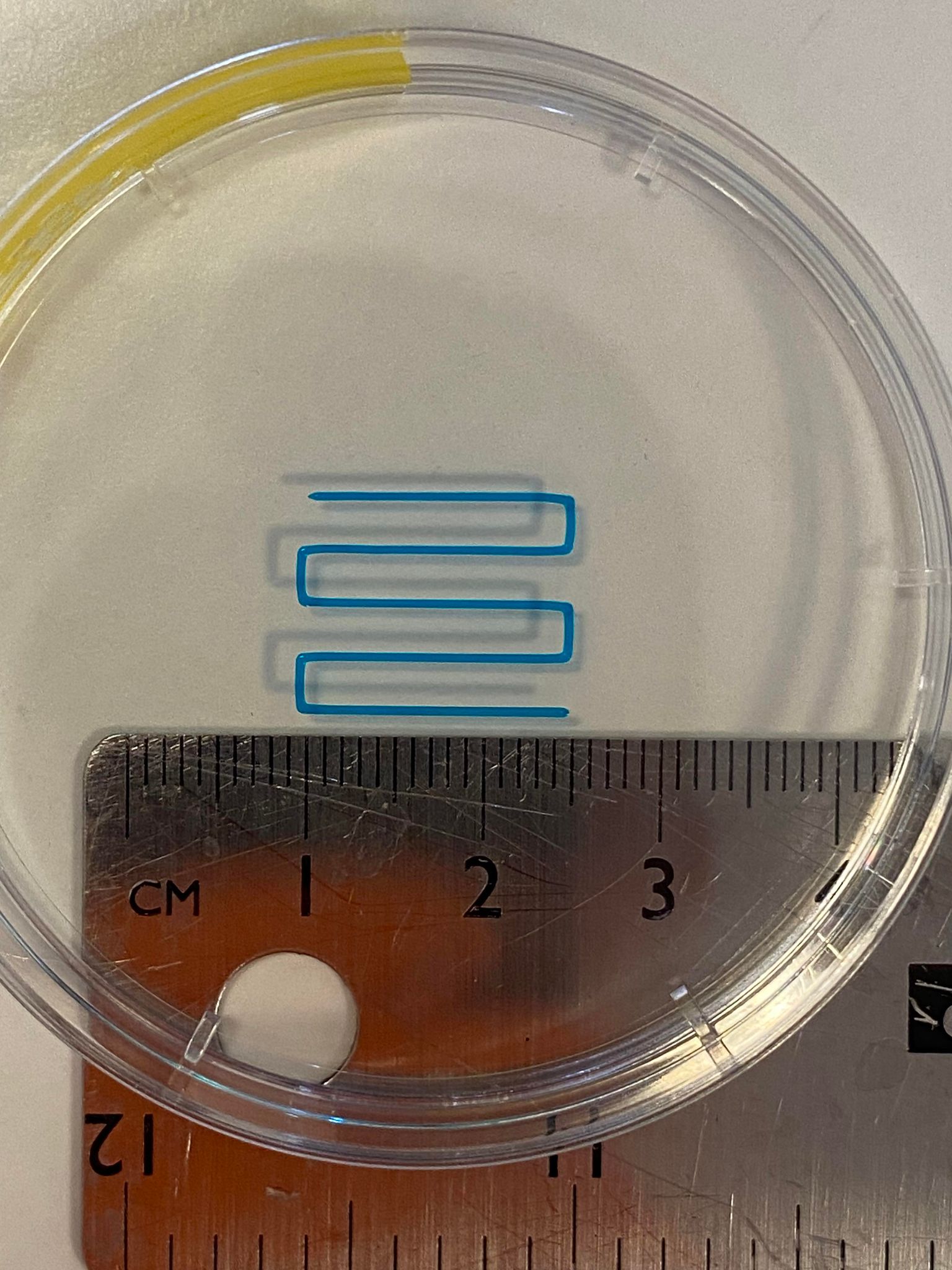

Supplement: S3 Data — (ZIP) [file pone.0312726.s005.zip › Figure 3 data/Filament printing/5.jpg]

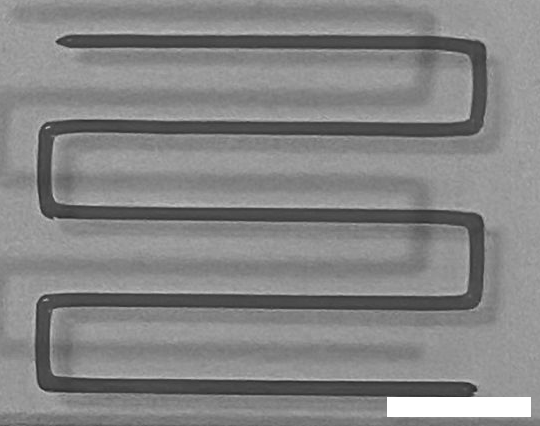

Supplement: S3 Data — (ZIP) [file pone.0312726.s005.zip › Figure 3 data/Filament printing/5mms-1 air. 60kpa 5um scale bar.png]

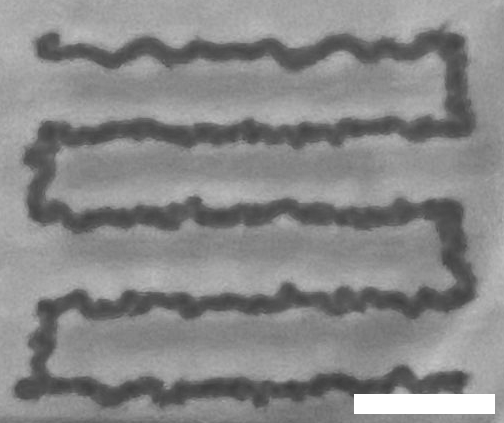

Supplement: S3 Data — (ZIP) [file pone.0312726.s005.zip › Figure 3 data/Filament printing/5mms-1 GG 60kpa 5um scale bar.png]

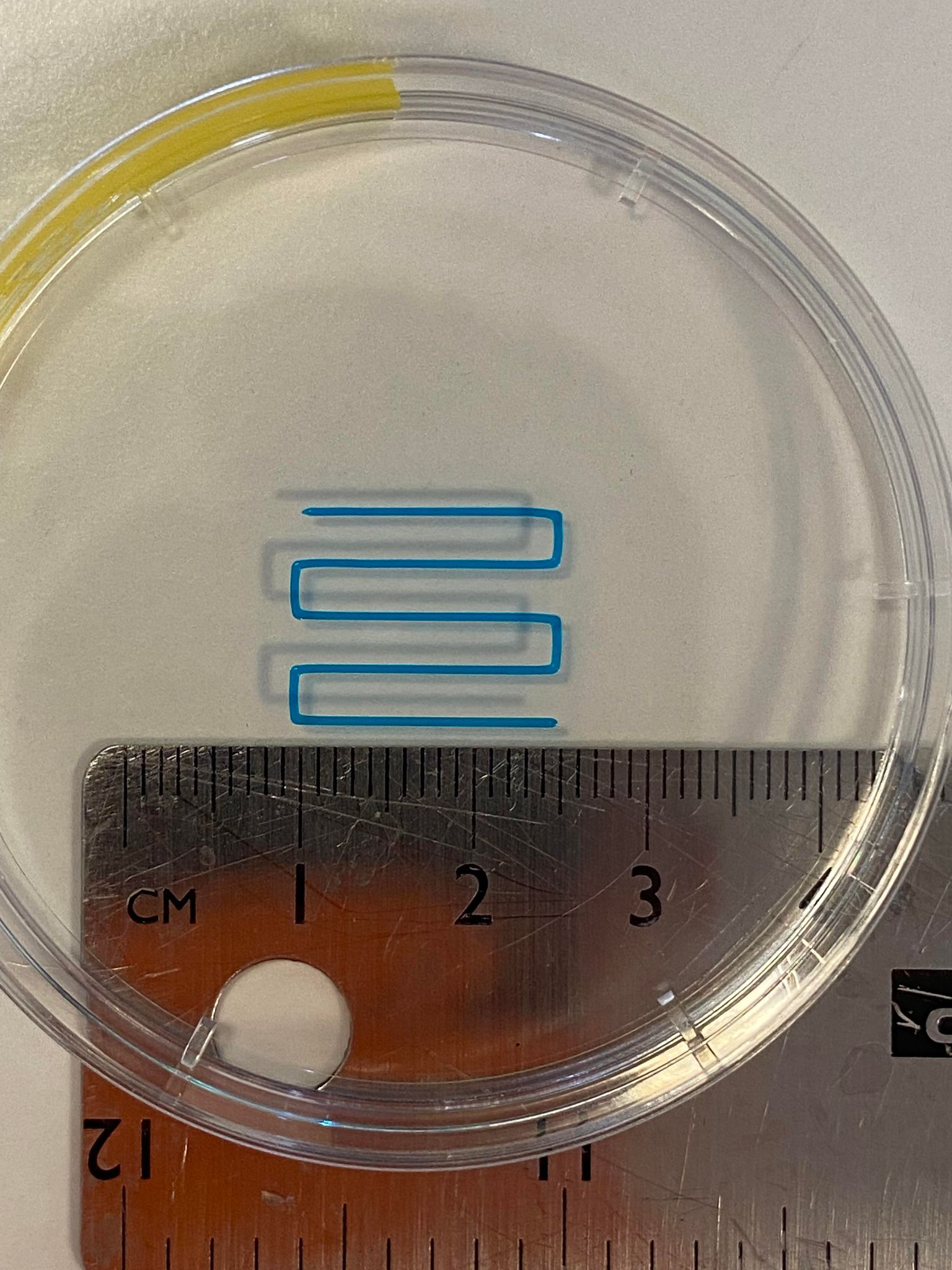

Supplement: S3 Data — (ZIP) [file pone.0312726.s005.zip › Figure 3 data/Filament printing/6.jpg]

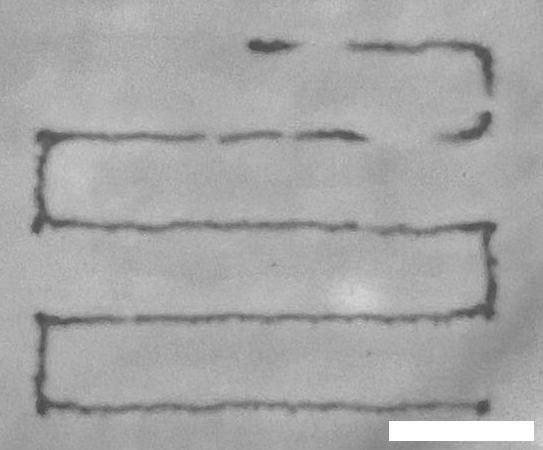

Supplement: S3 Data — (ZIP) [file pone.0312726.s005.zip › Figure 3 data/Filament printing/60mms-1 GG 60kpa 5um scale bar.png]

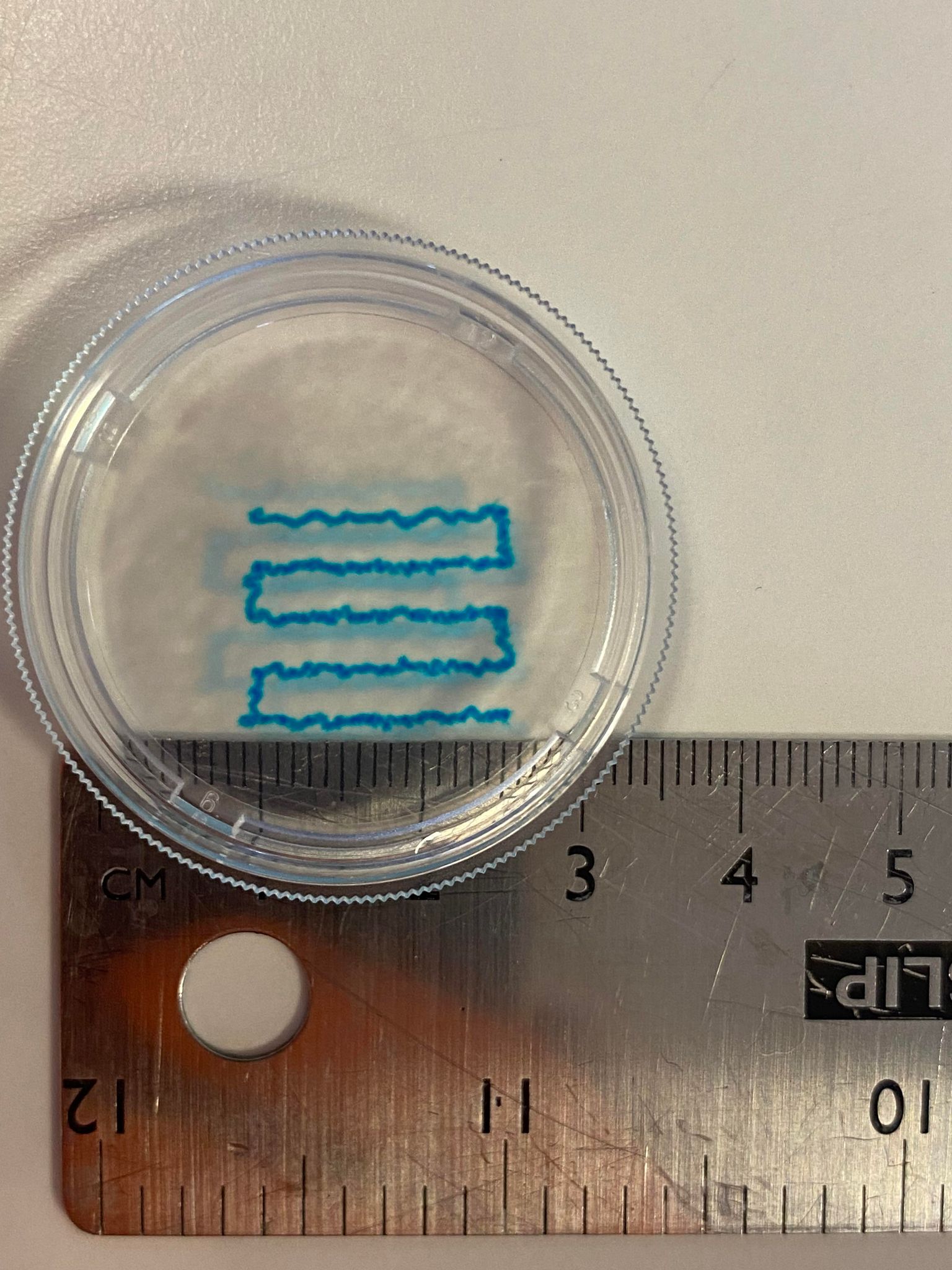

Supplement: S3 Data — (ZIP) [file pone.0312726.s005.zip › Figure 3 data/Filament printing/7.jpg]

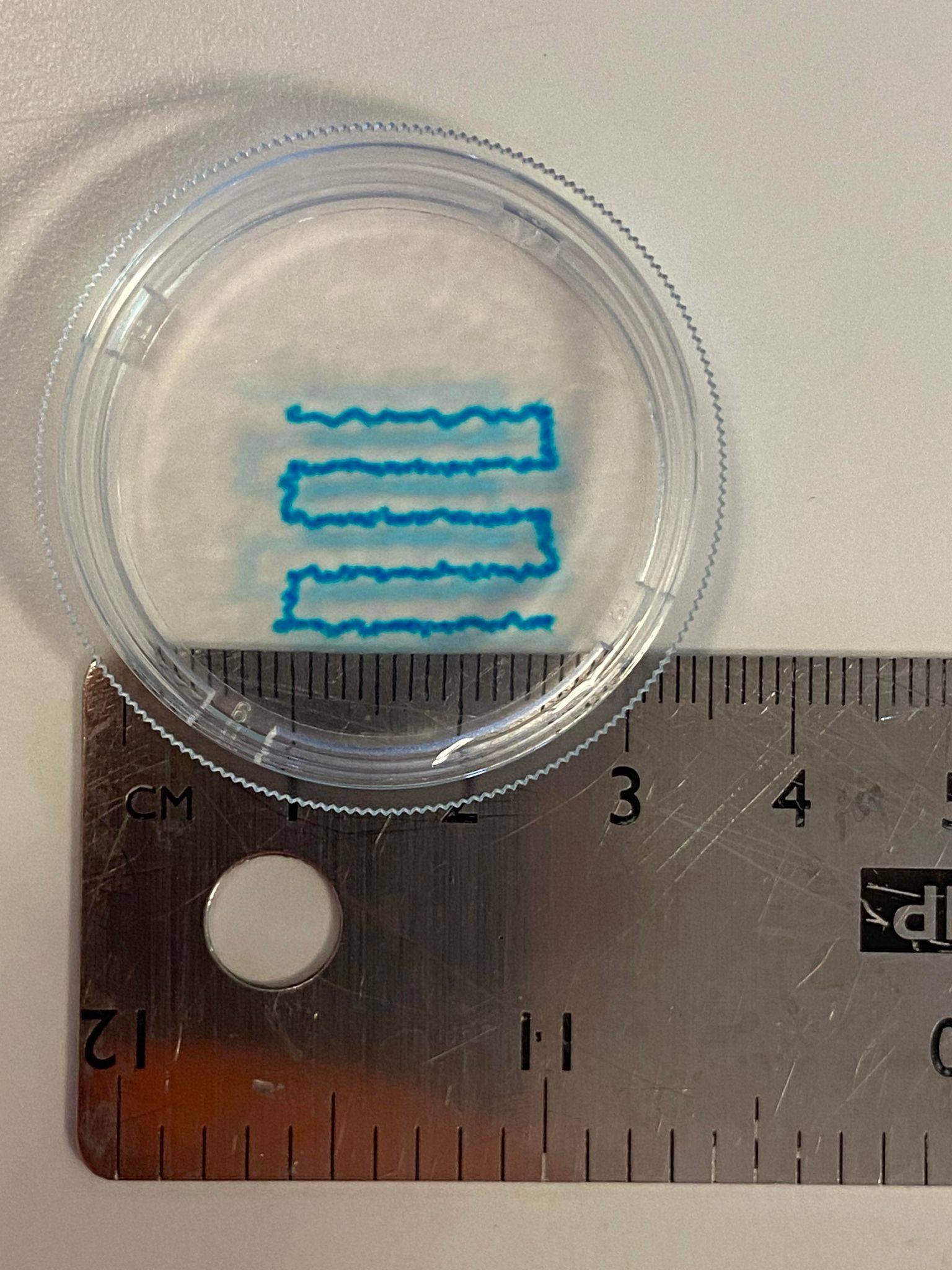

Supplement: S3 Data — (ZIP) [file pone.0312726.s005.zip › Figure 3 data/Filament printing/8.jpg]

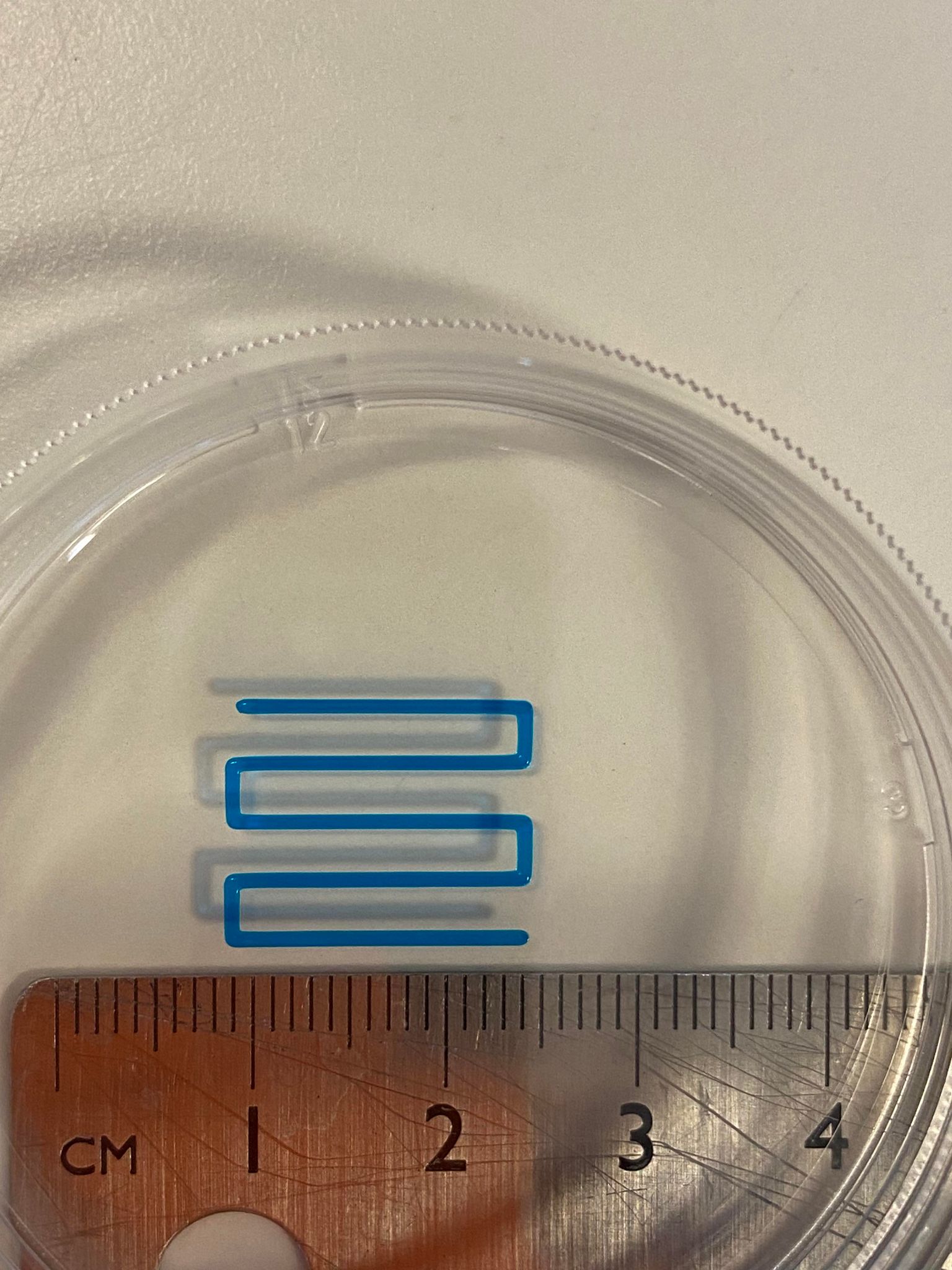

Supplement: S3 Data — (ZIP) [file pone.0312726.s005.zip › Figure 3 data/Filament printing/9.jpg]
